# Crystal Structures of Two Immune Complexes Identify Determinants for Viral Infectivity and Type-Specific Neutralization of Human Papillomavirus

**DOI:** 10.1128/mBio.00787-17

**Published:** 2017-09-26

**Authors:** Zhihai Li, Daning Wang, Ying Gu, Shuo Song, Maozhou He, Jingjie Shi, Xinlin Liu, Shuangping Wei, Jinjin Li, Hai Yu, Qingbing Zheng, Xiaodong Yan, Timothy S. Baker, Jun Zhang, Jason S. McLellan, Shaowei Li, Ningshao Xia

**Affiliations:** a State Key Laboratory of Molecular Vaccinology and Molecular Diagnostics, School of Life Sciences, Xiamen University, Xiamen, China; b National Institute of Diagnostics and Vaccine Development in Infectious Disease, School of Public Health, Xiamen University, Xiamen, China; c Department of Chemistry and Biochemistry; Division of Biological Sciences, University of California, San Diego, San Diego, California, USA; d Department of Biochemistry and Cell Biology, Geisel School of Medicine, Dartmouth College, Hanover, New Hampshire, USA; LCO/NCI/NIH; Johns Hopkins Bloomberg School of Public Health

**Keywords:** human papillomavirus, infectivity, neutralization, structure, type specificity

## Abstract

Persistent, high-risk human papillomavirus (HPV) infection is the primary cause of cervical cancer. Neutralizing antibodies elicited by L1-only virus-like particles (VLPs) can block HPV infection; however, the lack of high-resolution structures has limited our understanding of the mode of virus infection and the requirement for type specificity at the molecular level. Here, we describe two antibodies, A12A3 and 28F10, that specifically bind to and neutralize HPV58 and HPV59, respectively, through two distinct binding stoichiometries. We show that the epitopes of A12A3 are clustered in the DE loops of two adjacent HPV58 L1 monomers, whereas 28F10 recognizes the HPV59 FG loop of a single monomer. Via structure-based mutagenesis and analysis of antibody binding, we further identified the residues HPV58 D154, S168, and N170 and HPV59 M267, Q270, E273, Y276, K278, and R283, which play critical roles in virus infection. By substituting these strategic epitope residues into other HPV genotypes, we could then redirect the type-specific binding of the antibodies to these genotypes, thus highlighting the importance of these specific residues, HPV58 R161, S168, and N308 and HPV59 Q270, E273, and D281. Overall, our findings provide molecular insights into potential structural determinants of HPV required for infectivity and type specificity.

## IMPORTANCE

High-risk human papillomaviruses (HPVs) are considered the major causative pathogens of cancers that affect epithelial mucosa, such as cervical cancer. However, because of the lack of high-resolution structural information on the sites of neutralization, we have yet to determine the precise mode of HPV infection and how different types of HPV cause infection. Our crystal structures in this study have uncovered discrete binding stoichiometries for two different antibodies. We show that one A12A3 Fab binds to the center of one HPV58 pentamer, whereas five 28F10 Fabs bind along the top fringe of one HPV59 pentamer. Furthermore, through targeted epitope analysis, we show that 6 to 7 discontinuous residues of the L1 major capsid protein of HPV are determinants, at least in part, for virus infection and type specificity. This knowledge will help us to unravel the process of HPV infection and can potentially be used to drive the development of therapeutics that target neutralization-sensitive sites.

## INTRODUCTION

High-risk human papillomavirus (HPV) infection is the main cause of cervical and other anogenital cancers (1), with 15 HPV genotypes considered high risk (2). HPVs are nonenveloped, double-stranded DNA viruses that consist of multiple copies of the major (L1) and minor (L2) capsid proteins. Each of the 72 pentamers is composed of five copies of the L1 protein, which can self-assemble into an empty T = 7 icosahedral shell called a virus-like particle (VLP) (3). HPV L1 VLPs are excellent immune antigens for prophylactic vaccines (4–6), as they authentically resemble the native virion in capsid structure and immunological function (3, 7). During HPV VLP assembly, the minor protein L2 is dispensable (8).

Cryo-electron microscopy (cryo-EM) structures of the whole virus capsid have provided critical insight into the mechanism of HPV assembly (9–12), and crystal structures of the T = 1 L1-only VLP (HPV16) and L1 pentamers (HPV11, -16, -18, and -35) have illustrated how the HPV L1 monomer forms a canonical, eight-stranded β-barrel (BIDG-CHEF) joined by six highly variable loops (BC, CD, DE, EF, FG, and HI), five of which (all but CD) are located on the surface of the L1 pentamer (13, 14). Biochemical and serological assays have further revealed that the neutralizing epitopes for HPV capsids are type restricted and mainly clustered on these six hypervariable loops of L1 (15–20). These findings are supported by medium-resolution cryo-EM structures of HPV16 capsid in complex with neutralizing antibodies (21, 22).

The HPV capsid is purported to attach to the host cell primarily through heparan sulfate proteoglycans (HSPGs) and the non-HSPG extracellular matrix receptor, laminin-332 (formerly laminin-5), which is secreted by epithelial cells (23–26). L1 capsid undergoes conformational changes for downstream virus entry events, and studies have suggested that HPV antibodies could neutralize the virus by preventing these conformational changes (HPV16.V5 and HPV16.E70) (27) or by interfering with the virus-HSPG interaction (HPV16.U4) (28). HSPG has also been implicated as an attachment factor for other viruses, including the dengue virus (29), respiratory syncytial virus (30), and coxsackievirus (31). Cocrystal structures of HPV16 L1 bound with heparin (32, 33) revealed the requirement of multiple lysine residues (K54, K59, K278, K356, K361, K442, and K443) for virus binding to HSPG, whereas other studies have indicated the involvement of specific receptors that aid in HPV infection (34–40). Despite these findings, the molecular mechanism of virus infection remains elusive in part because of a lack of high-resolution structural information. Indeed, it has been suggested that the L1 surface loops of different HPVs exhibit a high degree of variation, and this variability is the main reason why there is limited cross-protection conferred by the current, approved VLP-based vaccines (14, 16, 18, 19, 41–44). Therefore, it is necessary to structurally determine the type-specific epitopes to understand the type specificity for HPV immunology.

Here, we characterize two type-specific, neutralizing monoclonal antibodies (MAbs), A12A3 and 28F10, against HPV58 and HPV59, respectively, and report the crystal structures of the HPV pentamers in complex with the antigen-binding fragments (Fabs) to resolutions of 3.5 and 3.4 Å, respectively. Through structural and biophysical data, we show that each antibody has a unique binding mode and stoichiometry. Fitting the Fab-bound pentamer crystal structures into corresponding medium-resolution cryo-EM structures of Fab-bound HPV L1 VLPs, we show that these binding modes are compatible with antibody recognition of whole virions. Furthermore, using site-directed mutagenesis, we identified the key epitope residues that are critical for binding type-specific antibodies. Functional studies showed that some residues located on the epitopes for both A12A3 and 28F10 are involved in virus infection and that type-specific antibody binding could be redirected to HPV33 and HPV18 L1 chimeric VLPs, respectively, after swapping key epitope residues. These findings narrow the functional surface area of HPV capsid to the key residues required for virus infection and offer molecular insight into the antibody-mediated HPV neutralization associated with type specificity.

## RESULTS

### Characterization of two HPV type-specific MAbs, A12A3 and 28F10.

**FIG 1:**
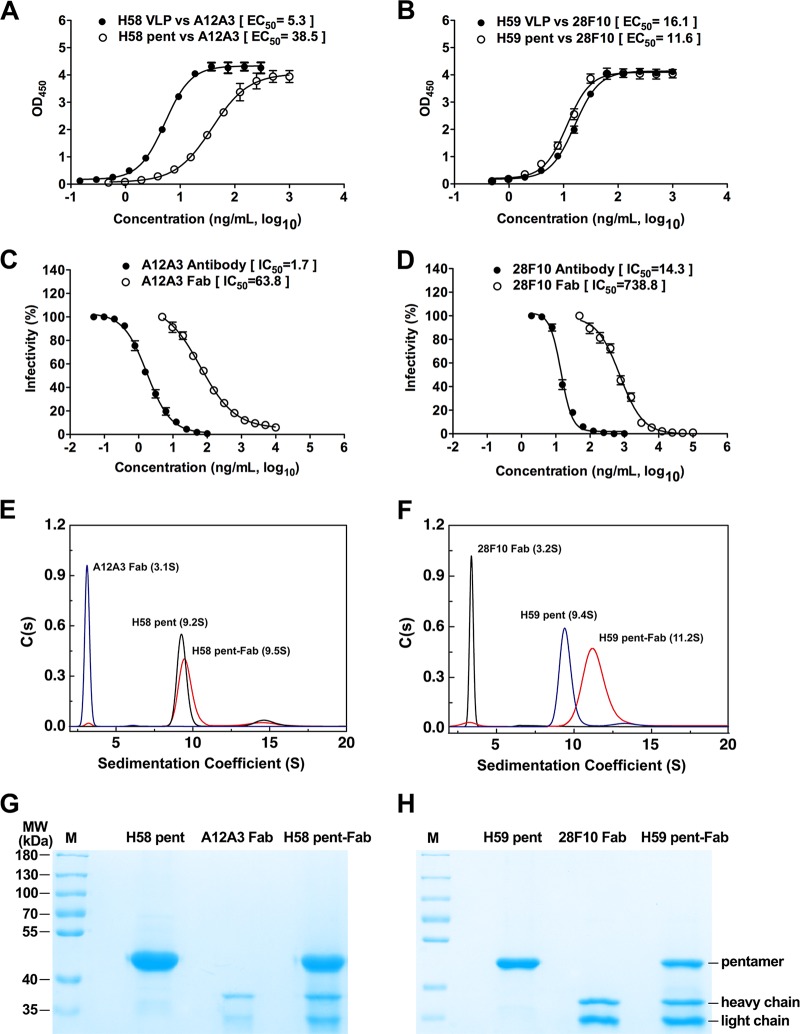
Binding of HPV58 and HPV59 pentamers to their type-specific neutralizing antibodies, A12A3 and 28F10, respectively. (A and B) Binding profiles of MAbs A12A3 and 28F10 with VLPs or pentamers of HPV58 (A) and HPV59 (B), respectively. (C and D) Neutralization of HPV pseudoviruses of types 58 and 59 by the MAb or Fab fragments of A12A3 (C) and 28F10 (D), respectively. (E and F) Sedimentation coefficient profiles of (E) HPV58 pentamer, A12A3 Fab, and their immune complexes and (F) HPV59 pentamer, 28F10 Fab, and their immune complexes. (G and H) AUC samples of HPV58 (G) and HPV59 (H) assessed by SDS-PAGE.

Two murine MAbs—A12A3 (IgG2b) and 28F10 (IgG2a)—were raised against HPV L1-only VLPs derived from HPV58 and HPV59, respectively (see Fig. S1A in the supplemental material). Using enzyme-linked immunosorbent assays (ELISAs) against 11 different HPV genotypes (HPV6, -11, -16, -18, -31, -33, -35, -45, -52, -58, and -59), we found that A12A3 and 28F10 antibodies exclusively recognized their corresponding genotypes, with high binding capacities for MAb A12A3 against HPV58 VLPs (ELISA titer, 10^4.3^; 50% effective concentration [EC_50_], 5.3 ng/ml) and MAb 28F10 against HPV59 VLPs (ELISA titer, 10^4.1^; EC_50_, 16.1 ng/ml) (Fig. 1A and B; Fig. S1A). The type specificities of A12A3 and 28F10 antibodies were further confirmed in pseudovirus (PsV)-based neutralization assays, and each MAb neutralized only the genotype that it was raised against (Fig. S1A).

10.1128/mBio.00787-17.1 FIG S1  Characterization of MAbs A12A3 and 28F10. (A) Type specificity of MAb 12A3 and 28F10. (B and C) Binding of MAb 4B3 to pentamer and VLPs of HPV58 (B) and HPV59 (C). 4B3 is a wide-spectrum linear antibody that was employed to normalize the concentration of pentamer and VLPs used for the binding assays against A12A3 and 28F10, respectively. (D) Representative negative images for both VLP-Fab and VLP-IgG complexes of HPV58:A12A3 and HPV59:28F10. The red arrows indicate the bound Fabs or IgGs. Images are shown to indicate the both A12A3 and 28F10 IgGs are capable of cross-linking HPV58 and HPV59 VLPs, respectively. Download FIG S1, TIF file, 2.5 MB. Copyright © 2017 Li et al. 2017 Li et al. This content is distributed under the terms of the Creative Commons Attribution 4.0 International license.

HPV L1 VLPs form by the assembly of 72 L1 pentamers, and these pentamers retain most of the type-specific neutralizing epitopes found on L1 VLPs and could induce a lower anti-HPV neutralization titer than VLPs (3, 45–47). Therefore, to understand the molecular basis for antibody-mediated neutralization of HPV, we investigated the interactions between the HPV pentamers and Fab fragments. First, we measured the binding capabilities of A12A3 and 28F10 IgGs to HPV58 and HPV59 pentamers (HPV58p and -59p), respectively, and compared them with binding to VLPs. We found that A12A3 IgG did not bind as well to HPV58p as it did to HPV58 VLPs (EC_50_, 38.5 ng/ml for pentamer and 5.3 ng/ml for VLPs [Fig. 1A; Fig. S1B]), whereas the binding profiles of 28F10 IgG to the pentamer and VLPs of HPV59 were similar (Fig. 1B; Fig. S1C). Using a neutralization assay, we found that the IC_50_s of A12A3 IgG and 28F10 IgG were 1.7 and 14.3 ng/ml, respectively, whereas those of the Fabs were 63.8 and 738.8 ng/ml, respectively. Thus, the IgGs of A12A3 and 28F10 are about 40- and 50-fold, respectively, more potent at neutralization than their corresponding Fabs in this assay (Fig. 1C and D). Electron microscopy observations of VLP:Fab and VLP:IgG of both HPV58:A12A3 and HPV59:28F10 show that both A12A3 and 28F10 IgGs could simultaneously capture two VLPs (Fig. S1D), indicating the discrepancy of neutralizing activity between Fabs and IgGs may be attributed to the avid binding of the IgGs with its two arms binding to different virus particles, which would produce a much higher apparent affinity than that of the monovalent Fabs. Another possible explanation is that the Fc portions of the IgGs may sterically impede attachment of the virions to host cell receptors during HPV infection.

Next, we incubated the HPVp and Fab fragments and purified them by gel filtration chromatography. This allowed us to obtain highly purified, homogeneous samples of the HPV58p:A12A3 and HPV59p:28F10 immune complexes for crystallization. Notably, in analytical ultracentrifugation (AUC) experiments, we obtained similar sedimentation coefficients for HPV58p and -59p (9.2S and 9.4S, respectively) and A12A3 and 28F10 Fabs (3.1S and 3.2S, respectively), but a much higher sedimentation coefficient for HPV59p:28F10 (11.2S) than for HPV58p:A12A3 (9.5S) (Fig. 1E and F). Similarly, SDS-PAGE analysis revealed a lower stoichiometry of A12A3 Fab to HPV58 L1 than that of 28F10 Fab to HPV59 L1 (Fig. 1G and H). These results suggest that the Fabs have distinct binding modes for the two HPV L1s.

### Crystal structures of HPV58p:A12A3 and HPV59p:28F10 reveal different binding modes of neutralizing antibodies on HPV pentamers.

**FIG 2:**
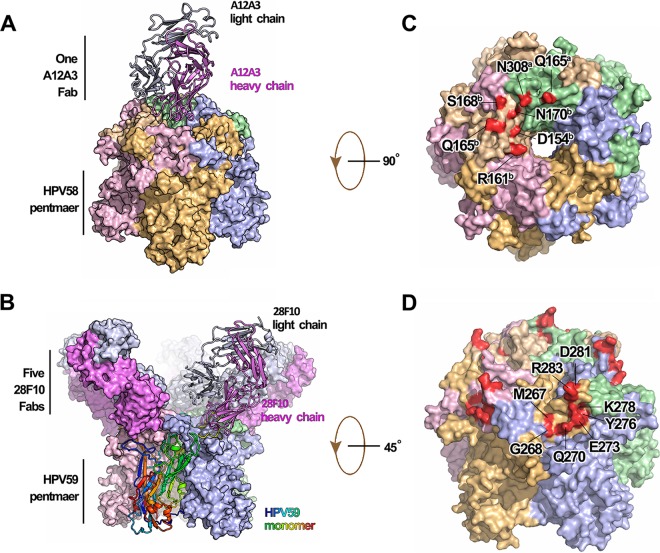
Crystal structures of the immune complexes of HPV58p:A12A3 and HPV59p:28F10. (A) HPV58p:A12A3 structure with the Fab shown as a ribbon and the antigen in surface representations. (B) HPV59p:28F10 structure with a monomer and its bound Fab shown as a ribbon and the monomer colored from blue to red (N terminus to C terminus). (C and D) The footprints of MAbs A12A3 (C) and 28F10 (D). Both immune complexes are represented in the same color scheme with different chains: chain a in pale green, chain b in wheat, chain c in light pink, chain d in light orange, and chain e in light blue for the HPV pentamer and the heavy chain in violet and the light chain in white-blue for antibody.

To understand how HPV type-specific antibodies bind to virions and inhibit HPV infection, we determined the crystal structures of HPV58p alone, as well as those of the HPV58p:A12A3 and HPV59p:28F10 immune complexes to resolutions of 2.0, 3.5, and 3.4 Å, respectively (Table 1 and Fig. 2; see Fig. S2A in the supplemental material). The structures were solved by molecular replacement and refined to *R*_work_/*R*_free_ values of 16.8%/19.8%, 20.2%/24.9%, and 23.0%/26.1%, respectively. In the overall structure of HPV58p:A12A3, we note that only one A12A3 Fab is bound to the center of the HPV58 pentamer at a 70° angle to the pentamer surface (Fig. 2A; Fig. S2B and S2D). This 1:1 Fab-pentamer binding pattern is similar to that of the H11.B2 antibody based upon a low-resolution cryo-EM structure (48). For HPV59p:28F10, five 28F10 Fabs are located around the periphery of the upper rim of the HPV59 pentamer (Fig. 2B), with a binding angle of about 54° to the pentamer surface (Fig. S2C). It should be noted that the asymmetric unit contains two HPV59p:28F10 complexes, each in the shape of a flower with 5 petals, wherein a 28F10 Fab from one complex inserts into the center of the second complex (Fig. S2E). As such, there are slightly different elbow angles among the 10 Fabs in the asymmetric unit (Fig. S2F). This 5:1 Fab-pentamer binding stoichiometry appears to be common and has been observed in other HPV type-specific antibody structures (21, 22). Collectively, the two distinct binding modes observed for these two Fabs provide a molecular basis for the differences observed in our AUC and SDS-PAGE results (Fig. 1E to H).

10.1128/mBio.00787-17.2 FIG S2  Crystal structure of HPV58 pentamer, HPV58p:A12A3 and HPV59p:28F10. (A) Crystal structure of HPV58 pentamer with five monomers in different colors: chain a (pale green), chain b (wheat), chain c (light pink), chain d (light orange) and chain e (light blue). (B and C) Binding configurations of HPV58p:A12A3 and HPV59p:28F10. MAbs A12A3 and 28F10 obliquely bind to the pentamer surface with tilt angles of 70° (B) and 54° (C), respectively. (D and E) Crystal packing of HPV58p:A12A3 and HPV59p:28F10 complexes. These two crystals have a P2_1_ space group. There are one HPV58p:A12A3 molecule (D) and two HPV59p:12F10 molecules (E) in one crystallographic asymmetric unit. The content equivalent to one crystal cell is presented in ribbon mode, the unique axis is drawn vertically in the figure to show the translation and screw symmetry of the P2_1_ space group. (F) Superimposition of 10 28F10 Fabs in one asymmetric unit. Superimposition of 10 28F10 Fabs shows the variability in the intertwined angle between the variation and constant regions due to flexibility of the hinge linking the V and C regions. Download FIG S2, TIF file, 2.3 MB. Copyright © 2017 Li et al. 2017 Li et al. This content is distributed under the terms of the Creative Commons Attribution 4.0 International license.

**TABLE 1:** X-ray data collection and refinement statistics

Parameter	Value(s) for^a^:		
	HPV58 L1	HPV58p:A12A3 Fab	HPV59p:28F10 Fab
Data collection			
Unit cell dimensions			
*a*, *b*, *c*, Å	187.2, 101.8, 136.2	121.6, 102.6, 138.0	116.3, 417.2, 134.9
α = β = γ, °	90.0, 95.7, 90.0	90.0, 114.5, 90.0	90.0, 110.0, 90.0
Space group	C2	P2_1_	P2_1_
Resolution range, Å	30.0–2.0 (2.08–2.04)	50.0–3.5 (3.51–3.44)	50.0–3.4 (3.42–3.36)
Wavelength, Å	0.9792	0.9795	0.9792
*hkl* (*I* > σ)			
Observed	1,112,559	255,089	523,015
Unique	159,674	41,030	161,106
Redundancy	7.0 (5.3)	6.2 (5.9)	3.2 (3.2)
Completeness, %	99.2 (91.0)	99.9 (99.9)	94.5 (94.1)
*I*/σ*I*	19.6 (2.4)	10.4 (2.1)	8.5 (1.6)
*R*_sym_, %^b^	11.3 (55.7)	26.0 (99.9)	14.4 (77.7)

Refinement			
Resolution range, Å	29.8–2.04	47.9–3.44	36.5–3.35
Reflections, no.	159,523	40,995	161,016
*R* factor, %^c^	16.8	20.2	23.0
*R*_free_, %^d^	19.8	24.9	26.1
RMSD			
Bond length, Å	0.002	0.002	0.003
Bond angle, °	0.53	0.51	0.65
Protein residues, no.	2,083	2,493	8,582
B factor, Å^2^			
Wilson	30.9	77.3	77.6
Avg	47.8	98.5	96.0
Ramachandran plot, %			
Favored	96.8	94.1	92.6
Allowed	3.2	5.1	6.8
Outliers	0.0	0.8	0.6

a Numbers in parentheses refer to the highest-resolution shell.

b *R*_sym_ = Σ_*h*_ Σ_*i*_|*I*_*1*_(*h*) − <*I*(*h*)|/Σ_*h*_ Σ_*i*_ *I*_*1*_(*h*).

c *R* factor = Σ_*hkl*_||*F*_obs_| − *k*|*F*_calc_||/Σ_*hkl*_|*F*_obs_|.

d *R*_free_ was calculated using the same equation for the *R* factor, but 5.0% of reflections were chosen randomly and omitted from the refinement.

Further structural analysis of the two immune complexes revealed shared characteristics. First, we observed that the heavy chain of the antibody dominated binding to the antigen. The interactions between the HPV58p and A12A3 Fab buried a total of 846 Å^2^ of surface area, as calculated by PISA (49), with about 80% (679 Å^2^) buried by the heavy chain. Similarly, the heavy chain buried 82% of the total buried surface area in the HPV59p:28F10 complex (625 of 725 Å^2^).

**FIG 3:**
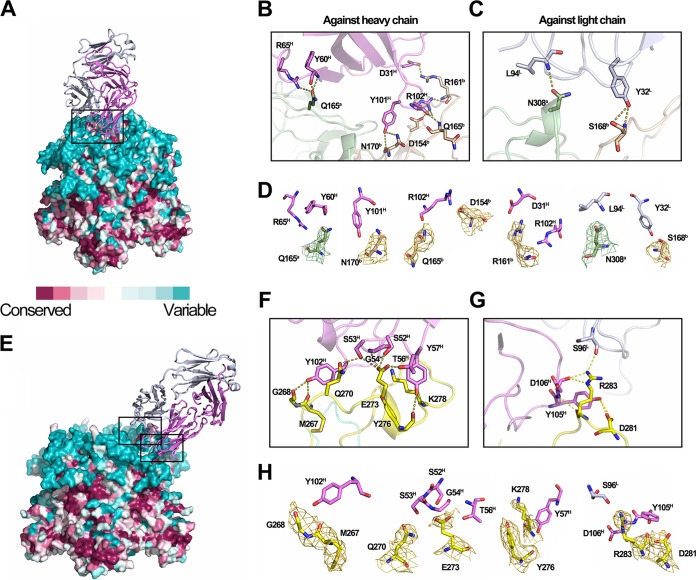
Antigen-antibody interactions in the HPV58p:A12A3 and HPV59p:28F10 complexes. (A and E) Surface representations of HPV58 and -59 pentamers (HPV58p and -59p) colored as per the sequence conservation, which is based on alignment of multiple HPV L1 sequences (see Fig. S8 in the supplemental material). In panel E, four of the binding 28F10 Fabs are omitted for clarity. Close-up views of the interfaces are shown in panels B and C for HPV58p:A12A3 and panels F and G for HPV59p:28F10. (B and C) The interactions of HPV58 against the heavy chain (B) and light chain (C) of MAb A12A3 are shown. (F and G) Magnified views of the upper (F) and lower (G) boxed regions in panel E. Side chains involved in the interaction between antigen and antibody are labeled and shown as sticks. (D and H) All contacts are depicted as dotted lines. Individual contacts in the interface of HPV58p:A12A3 (D) and HPV59p:28F10 (H) are displayed with the sample electron density (2Fo-Fc) maps contoured at 1σ above the mean shown for the epitope residues of HPV.

In the HPV58p:A12A3 structure, the antibody binds across two adjacent HPV58 monomers—a and b—with six residues in the DE loops of different monomers (Q165^a^, D154^b^, R161^b^, Q165^b^, S168^b^, and N170^b^), and one residue (N308^a^) in the FG loop of monomer a (Fig. 2C and Fig. 3A to D; see Table S1A in the supplemental material). Particularly, 12 interactions mediate recognition between the antigen (HPV58 pentamer) and antibody (A12A3), comprising 2 salt bridges (D154^b^-R102^H^ and R161^b^-D31^H^ [Fig. 3B; Table S1A]) and 10 hydrogen bonds involving both the main and side chains (Q165^a^-R65^H^, Q165^a^-Y60^H^, R161^b^-D31^H^, R161^b^-R102^H^, Q165^b^-R102^H^, N170^b^-Y101^H^, S168^b^-Y32^L^, and N308^a^-L94^L^ [Fig. 3B to D; Table S1A]). In the HPV59p:28F10 structure, all eight residues (M267, G268, Q270, E273, Y276, K278, D281, and R283) involved in binding to 28F10 are located on the FG loop of a single monomer (Fig. 2D and Fig. 3E to H; Table S1B) and are involved in 15 hydrogen bonds (Fig. 3F to H; Table S1B). Among these contacts, both Q270 and R283 form four hydrogen bonds with the antibody, suggesting critical roles for these 2 amino acids (aa) in this interaction (Fig. 3G; Table S1B). Only one residue from the light chain (S96^L^) was involved in the binding interface (Fig. 3G).

10.1128/mBio.00787-17.9 TABLE S1 Interface analysis of the crystal structures of HPV58p:A12A3 (S1A) and HPV59p:28F10 (S1B). Download TABLE S1, DOCX file, 0.1 MB. Copyright © 2017 Li et al. 2017 Li et al. This content is distributed under the terms of the Creative Commons Attribution 4.0 International license.

### Cryo-EM structures of HPV VLP immune complexes demonstrate consistent antibody-binding modes for HPV capsids and pentamers.

**FIG 4:**
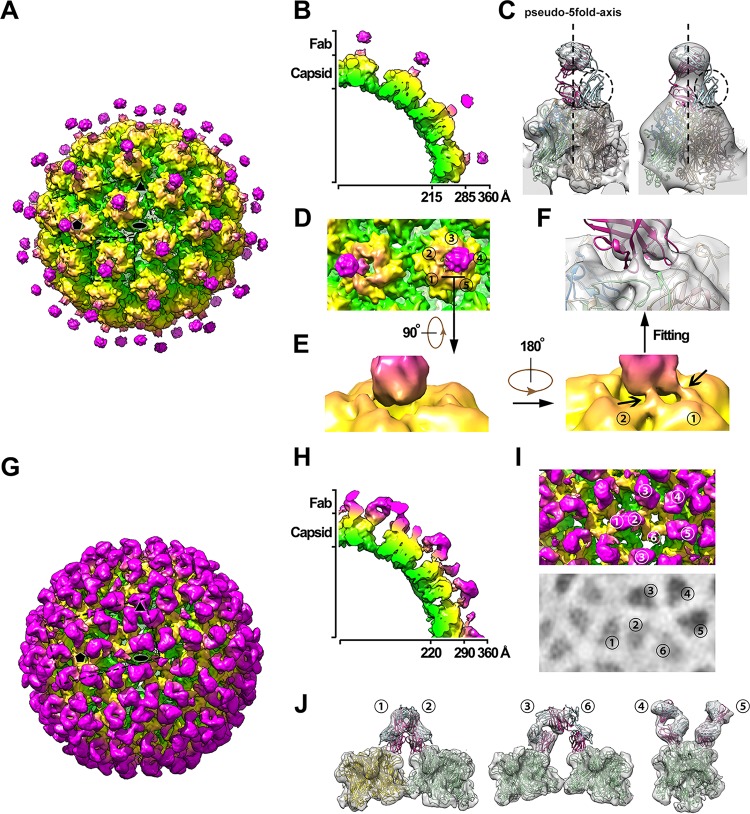
Cryo-EM structures of the VLP-Fab complexes of HPV58v:A12A3 and HPV59v:28F10. (A and G) Overall density maps of HPV58v:A12A3 (A) and HPV59v:28F10 (G) colored by radius from green (230 Å), to yellow (280 Å), to hot pink (320 Å). Icosahedral 2-, 3-, and 5-fold axes are indicated by black symbols. (B and H) Cross-sections of one-quarter of the cryo-EM map of HPV58-A12A3 (B) and HPV59-28F10 (H). (C) Density corresponding to one pentamer complexed with A12A3 Fab and the fitted crystal structure of HPV58p:A12A3. The 25-Å low-pass filtered map is shown on the right. The pseudo-5-fold axis is represented by a dotted line, and the broken circles outline the region in the crystal structure where the attributed density was missing. (D) Magnified view of the boxed area in panel A, including the 5-coordinated pentamer and one of its neighboring 6-coordinated pentamers. The number denotes five different monomers of the 6-coordinated pentamer. (E) Close-up view of the interaction region between A12A3 and HPV58 capsid denoted in panel D. (F) The same density in panel E is shown in semitransparent gray and superimposed with the crystal structure of HPV58p:A12A3. (I) Magnified view of the boxed area in panel G. The number indicates six different monomers within one icosahedral asymmetric unit. The lower panel shows the radial projection of the complex map at 310-Å radii, which indicates the density intensities on the Fabs bound to different monomers. (J) The density map of a pentamer with different Fabs in an icosahedral asymmetric unit was extracted and is superimposed with the crystal structure of HPV59p:28F10.

We next determined the cryo-EM structures of A12A3 Fab and 28F10 Fab in complex with HPV58 L1 VLPs (HPV58v:A12A3) and HPV59 L1 VLPs (HPV59v:28F10), respectively, to show congruency in the binding modes for the crystal structures and the capsid immune complexes. From the cryo-EM micrographs, we observed visible protrusions for both VLP-Fab complexes, which should be considered the bound Fabs (see Fig. S3A and B in the supplemental material). The cryo-EM structures of HPV58v:A12A3 and HPV59v:28F10 were reconstructed to 9.5 and 8.4 Å, respectively (Fig. 4A and G; Fig. S3C to E). The HPV58v:A12A3 cryo-EM structure showed two disconnected densities vertically bound to the central region of each pentamer on the HPV58 capsid (Fig. 4A and B). The density map of the capsid complex was reconstructed by applying an icosahedral symmetry operation, in which the whole Fab bound to the 6-coordinated pentamer is harbored in one asymmetric unit and only one-fifth of the Fab bound at the 5-fold icosahedral axis is included in the symmetry operation. Fitting the HPV58p:A12A3 crystal structure into the cryo-EM map revealed consistency between the Fab binding orientations in the pentamer and the capsid complexes. In the cryo-EM map, the Fab density is weak, especially in those regions attributed to the off-pseudo-5-fold-axis regions of Fab, including most of the variable domain of the light chain and the elbow-like linker between the variable and constant domains (Fig. 4C, left). The density remained low after low-pass filtering the map to 25 Å (Fig. 4C, right), indicating that the missing density was not caused by the flexibility of the corresponding regions.

10.1128/mBio.00787-17.3 FIG S3  Cryo-EM structures of VLP-Fab complexes of HPV58v:A12A3 and HPV59v:28F10. (A-B) Representative micrographs of HPV58V:A12A3 (A) and HPV59V:28F10 (B). (C) Resolution evaluation of the cryo-EM reconstructions by Fourier shell correlation (FSC) at criterion 0.143. (D and E) Structural features of the L1 monomer with the fitted model in pink extracted from the density maps of HPV58V:A12A3 (D) and HPV59V:28F10 (E). Download FIG S3, TIF file, 2.4 MB. Copyright © 2017 Li et al. 2017 Li et al. This content is distributed under the terms of the Creative Commons Attribution 4.0 International license.

In the crystal structure of HPV58p:A12A3, there could be five potential binding sites for A12A3 for each pentamer on the HPV58 capsid. Therefore, if the Fab bound to the pentamer in a random 5-fold orientation in each asymmetric unit of the capsid (one 6-coordinated pentamer and one monomer from its neighboring 5-coordinated pentamer), the nonoverlapping densities between Fabs within different capsid asymmetric units could be averaged during icosahedral reconstruction. This may be one possible explanation for the mismatch between the atomic model and the density map. If the contour level of the density map was adjusted to 2σ, we see two rope-like densities connecting the Fab with monomers 1 and 2 in the 6-coordinated pentamer (Fig. 4D and E; monomers are denoted as loci 1 to 5), which therefore demonstrates that the antigen recognized by MAb A12A3 on the 6-coordinated pentamer is dominated by a specific orientation among the five random binding directions. This illustrates a consistent binding pattern for MAb A12A3 to the pentamer and capsid of HPV58. The A12A3 epitopes on HPV58 are clustered into two regions of two adjacent HPV monomers (Fig. 2C and Fig. 3B and C), as confirmed by the excellent fitting of the crystal structure into the density map of the capsid complex (Fig. 4F).

The HPV59v:28F10 complex map shows the density corresponding to a bound 28F10 Fab at each of the 360 potential Fab binding sites on the HPV59 capsid (Fig. 4G and H). However, similar to the cryo-EM structure of H16.V5 complexed with HPV16 pseudovirus (22), the HPV59v:28F10 density map shows various binding occupancies for the six 28F10 Fabs (loci 1 to 6) bound to six different monomers within one asymmetric unit of the capsid; this could be a result of steric hindrance among bound Fabs (Fig. 4I and H). Fitting the crystal structure of the HPV59p:28F10 into this map, we found that Fab-1 competed for binding to the 5-coordinated pentamer with the Fab-2 bound to an adjacent 6-coordinated pentamer (Fig. 4J, left) and found a steric clash between Fab-3 and Fab-6, which stems from two neighboring 6-coordinated pentamers related to the same 5-coordinated pentamer (Fig. 4J, middle). As expected, the excellent fitting of Fab-4 and Fab-5 into the cryo-EM density map demonstrated the consistent binding pattern between 28F10 to the pentamer and the capsid (Fig. 4J, right). Moreover, in evaluating the density intensities corresponding to all six 28F10 Fab molecules, we propose that, in the initial binding to HPV59, 28F10 antibody can (i) freely attach to locations 4 and 5, as indicated by the strong densities for Fab-4 and -5, (ii) randomly bind to locations 1 and 2 with similar probabilities, and (iii) preferably bind to location 3 when competing with location 6, as shown by the much higher density for Fab-3 than for Fab-6 (Fig. 4J; see Fig. S4 in the supplemental material). In addition, we attempted to determine whether the presence of multiple binding sites on each pentamer on HPV virus may facilitate simultaneous engagement of both arms of the IgGs. Intriguingly, there are two putative models suitable for one 28F10 antibody to bind bivalently to a single capsid (Fig. S4E to G), which presents another explanation for its higher affinity than 28F10 Fab (Fig. 1C): however, no reasonable model can support two Fab arms of one A12A3 antibody binding to the same particle (Fig. S4B to D).

10.1128/mBio.00787-17.4 FIG S4  Binding configuration of 28F10 Fabs bound to HPV59 and the models to predict the bivalent IgG binding. (A) Relative mean intensities of the six 28F10 Fabs bound on an icosahedral asymmetric unit of the HPV59 capsid. The intensities of these six Fabs were normalized by the mean intensity of Fab-5, which has the strongest density among the bound Fabs. The densities of different Fabs were segmented, and their intensities were calculated individually using Chimera (69). (B and E) Overall density maps of HPV58v:A12A3 (B) and HPV59v:28F10 (E). The two neighboring pentamers for both maps are outlined. (C and D) The density maps of two pairs of pentamers with A12A3 Fabs were extracted and are superimposed with the crystal structure of HPV58p:A12A3. (F and G) The density maps of two pentamers with different Fabs in an icosahedral asymmetric unit were extracted and are superimposed with the crystal structure of HPV59p:28F10. The numbering of 28F10 Fabs is the same as in Fig. 4. The schematic representations of Fabs binding on pentamers for panels C and D and F and G are shown on the right. Download FIG S4, TIF file, 2.7 MB. Copyright © 2017 Li et al. 2017 Li et al. This content is distributed under the terms of the Creative Commons Attribution 4.0 International license.

### Identification of critical epitope residues.

**FIG 5:**
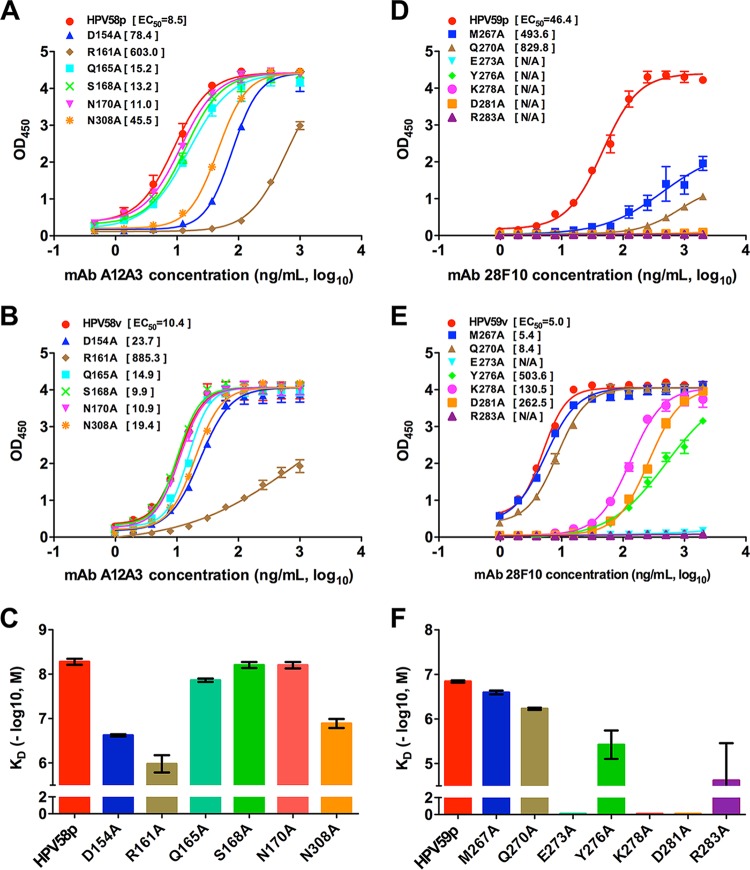
Effect of the interface residues on binding of MAbs A12A3 and 28F10. (A and D) Binding profiles of MAbs A12A3 and 28F10 to the mutant and WT HPV58 pentamer (A) and HPV59 pentamer (D), respectively. (B and E) Binding profiles of MAbs A12A3 and 28F10 to the mutant and WT HPV58 VLP (B) and HPV59 VLP (E), respectively. (C and F) Affinity constants of MAbs A12A3 and 28F10 to the mutant and WT HPV58 pentamer (C) and HPV59 pentamer (F), respectively. The vertical axis represents −log_10_ molar concentration of the equilibrium dissociation constant (*K*_*D*_). The color schemes for trace curves and histograms within HPV58 or HPV59 are the same.

In the crystal structures of HPV58p:A12A3 and HPV59p:28F10, several discontinuous amino acids at the interfaces of HPV were observed to interact with the antibodies (Table S1A and S1B). To ascertain which residues are critical for antibody recognition, we performed alanine-scanning mutagenesis on pentamers and VLPs. Six HPV58 residues (D154, R161, Q165, S168, N170, and N308) and seven HPV59 residues (M267, Q270, E273, Y276, K278, D281, and R283) were substituted for with alanine, and the resultant binding capacities of these variants were evaluated by indirect ELISA. We found that HPV58p bearing an R161A mutation (HPV58p R161A) showed dramatically lower binding reactivity to A12A3 than the wild-type (WT) HPV58p, with a more than 70-fold increase in the EC_50_ (603.0 ng/ml for R161A versus 8.5 ng/ml for WT) (Fig. 5A). The same mutation in the VLPs (HPV58v R161A) gave a comparable 80-fold higher EC_50_ (Fig. 5B). Mutations in the pentamer at D154A and N308A showed significantly decreased reactivity with A12A3, with a 5- to 9-fold increase in the EC_50_; however, these mutations in the VLP had just slightly lower A12A3 reactivity than the WT HPV58 VLP (2-fold EC_50_ increment). Mutations to the three other sites (Q165, S168, and N170) produced no significant effect on antibody binding to either the pentamer or VLP (Fig. 5A and B). Biacore binding affinity measurements for HPV58 showed that the pentamer variants D154A, R161A, and N308A had a >10 fold lower affinity than the WT pentamer or the other variants (Fig. 5C), consistent with the ELISA results. Moreover, the decrease in binding because of the mutations is a result of a faster dissociation rate in the surface plasmon resonance (SPR) curves (Fig. S5A; see Table S2 in the supplemental material), indicating that the side chains of D154, R161, and N308 facilitate the stability of the antigen-antibody complex.

10.1128/mBio.00787-17.5 FIG S5  Binding curves by surface plasma resonance (SPR) of MAbs A12A3 and 28F10 against HPV58 pentamer mutants (A) and HPV59 pentamer mutants (B), respectively. Five concentrations of HPV58 pentamer and HPV59 pentamer were injected onto a MAb A12A3-bound chip and a MAb 28F10-bound chip, respectively. These binding curves were used to calculate the affinity constants. The experiments were repeated twice, and the results were consistent. Download FIG S5, TIF file, 0.9 MB. Copyright © 2017 Li et al. 2017 Li et al. This content is distributed under the terms of the Creative Commons Attribution 4.0 International license.

10.1128/mBio.00787-17.10 TABLE S2 Binding kinetics of MAb A12A3 against HPV58 pentamer and MAb 28F10 against HPV59 pentamer. Download TABLE S2, DOCX file, 0.1 MB. Copyright © 2017 Li et al. 2017 Li et al. This content is distributed under the terms of the Creative Commons Attribution 4.0 International license.

For MAb 28F10, all seven alanine replacements in the HPV59 pentamers affected the binding, with E273A, Y276A, K278A, D281A, and R283A mutations completely abrogating the interaction. In contrast, HPV59p M267A and Q270A showed reduced binding capacity, with approximately 10- and 20-fold increases in the EC_50_, respectively (Fig. 5D). For HPV59 VLPs, the E273A and R283A mutations abolished 28F10 binding, whereas HPV59v Y276A, K278A, and D281A variants retained the antibody binding but showed significantly lower reactivity than the WT HPV59 VLP (Fig. 5E). Intriguingly, M267A and Q270A showed comparable 28F10 reactivities with HPV59 VLP. From these HPV59 binding assays, we show that (i) Y276A and R283A mutations cause a 10- to 100-fold decrease in affinity, (ii) E273A, K278A, and D281A have no binding signal in the SPR experiments, and (iii) M267A and Q270A exhibit slightly lower affinity than the WT HPV59 pentamer (Fig. 5F; see Fig. S5B and Table S2 in the supplemental material).

Interestingly, among the residues that affected binding to 28F10, the side chains of residues M267, Y276, and K278 did not interact with 28F10 in our crystal structure (Table S2). This may suggest that the long side chains of these three residues possibly contribute to the stability of the functional FG loop. This binding discrepancy and those for the point mutations between the pentamer and VLPs may indicate that conformational changes occur at the loop regions during the particle assembly process for both HPV59 and HPV58.

Collectively, site-directed mutagenesis revealed the residues HPV58 R161, HPV59 E273, and HPV59 R283 play essential roles in the interaction between the antigens and their corresponding antibodies. This is consistent with our structural observations that show that R161 in HPV58 forms two close contacts (2.7 and 2.9 Å, respectively) with A12A3, including one salt bridge (Table S1A), E273 in HPV59 is responsible for the two close contacts with 28F10 (2.3 and 2.5 Å), and R283 is the only residue in contact with both heavy and light chains of the antibody (2.8 and 3.2 Å, respectively) (Table S1B).

### Role of the epitopes of A12A3 and 28F10 in viral infectivity.

To verify the antibody-binding regions associated with the virus infection sites, we generated virus mutants of HPV58 and HPV59 using alanine substitutions for interface amino acids of HPV L1 involved in the interactions with MAbs A12A3 and 28F10, respectively. Here, we took advantage of an HPV pseudovirus (PsV), the shell of which consists of the L1 and L2 capsid proteins but which also carries a green fluorescent protein (GFP) marker genome instead of the viral genome. This allowed us to measure the infection of all mutant PsVs by visually counting the proportion of GFP-expressing cells at 72 h postinfection (50). The formation of intact particles for mutant PsVs was confirmed by electron microscopy (see Fig. S6E and S6F in the supplemental material). The input for the infection assay for all PsV mutants was normalized by the presence of conformational neutralizing epitopes for L1 using a combination of monoclonal antibody-based sandwich ELISA and Western blotting (Fig. S6A to D).

10.1128/mBio.00787-17.6 FIG S6  Characterization of WT and mutant pseudoviruses. (A and B) the presence of conformationally intact L1 proteins of mutant PsVs for infection assays was normalized to that of WT HPV58 (A) and HPV59 (B) using a conformation-dependent MAb-based sandwich ELISA. (C and D) L1-specific Western blotting was employed to confirm that all mutant virions of HPV58 (C) and HPV59 (D) contain L1 proteins in amounts comparable to those of the WT virions. (E and F) Negative electron micrographs of WT and mutant PsVs of HPV58 (E) and HPV59 (F). All of the inspected samples were used for the infection assays. Download FIG S6, TIF file, 2.5 MB. Copyright © 2017 Li et al. 2017 Li et al. This content is distributed under the terms of the Creative Commons Attribution 4.0 International license.

**FIG 6:**
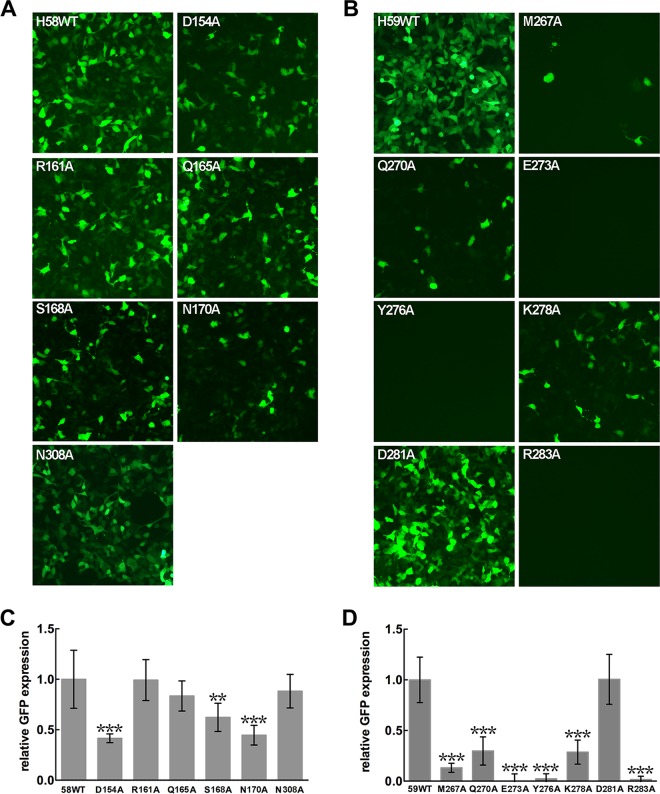
Infectivity of mutant pseudoviruses (PsVs). (A and B) HaCaT cells were infected with equal amounts of WT and mutant PsVs of HPV58 (A) and HPV59 (B). Expression of GFP was visualized using a fluorescence microscope. Panels C and D demonstrate the fluorescence intensities in panels A and B, respectively. Intensities were quantified from 10 randomly selected views using ImageJ, and the relative expression of the mutant PsVs to the cells infected with WT PsVs is plotted as the mean ± standard deviation (SD). Results were analyzed by unpaired Student’s *t* test. Differences were considered statistically significant at *P* ≤ 0.05 (not shown), *P* ≤ 0.01 (**), or *P* ≤ 0.0001 (***).

In HaCaT cells, we found that D154A and N170A mutations in HPV58 PsVs notably impaired their infectivity by more than half (Fig. 6A and C), suggesting the involvement of these residues in the infection process. The S168A mutant showed a reduction in infection ability by almost 30% compared with the WT HPV58 PsV. Except for the D281A virus, all of the mutant viruses of HPV59 showed significantly reduced infectivity in HaCaT cells (Fig. 6B and D), particularly the E273A, Y276A, and R283A viruses, which almost failed to infect the cells. In addition, the M267A, Q270A, and K278A viruses showed minimal infectivity. Taken together, we suggest that all six residues in the conformational epitope that binds to MAb 28F10 play a key role in mediating virus entry into the host cells. It is noteworthy that among these key sites for HPV59 infection, the analogous residues in HPV16 L1 (N270 and K278) and HPV18 L1 (Q273 and K278) are involved in binding to the cell-surface receptor heparin oligosaccharide (33) (see Fig. S7D and E in the supplemental material). Thus, we propose that MAb 28F10 neutralizes the virus by blocking its interaction with cell surface receptors.

10.1128/mBio.00787-17.7 FIG S7  Analysis of key sites of HPV59 (A and B) and HPV58 (C to E) involved in the virus infection procedure. (A and B) Structural comparison of heparin binding sites in HPV16, HPV18, and HPV59. (A) L1 monomer in HPV59p:28F10 (wheat) is superimposed with the crystal structures of HPV16 (pink) and HPV18 (light blue) pentamers bound to heparin (33) (PDB code: 3OAE for HPV16-heparin and 3OFL for HPV18-heparin). (B) Close-up view of the boxed region in panel A. HPV59 Q270, HPV59 E273, and HPV59 K278 are two analog residues with HPV16 N270, HPV18 Q273, and HPV16 K278/HPV18 K278, respectively, both of which are involved in binding to heparin. (C) Top view of the central region of HPV58 pentamer in the immune complex. The three A12A3 epitope residues that affected infectivity of HPV58 PsVs are shown in stick style and colored as per the scheme in Fig. 3 for the different monomers. (D) Structural alignment of five monomers. The central hollow channel side is labeled. (E) Close-up view of the boxed region in panel B. Download FIG S7, TIF file, 2.6 MB. Copyright © 2017 Li et al. 2017 Li et al. This content is distributed under the terms of the Creative Commons Attribution 4.0 International license.

### Antibody epitopes determine type specificity.

**FIG 7:**
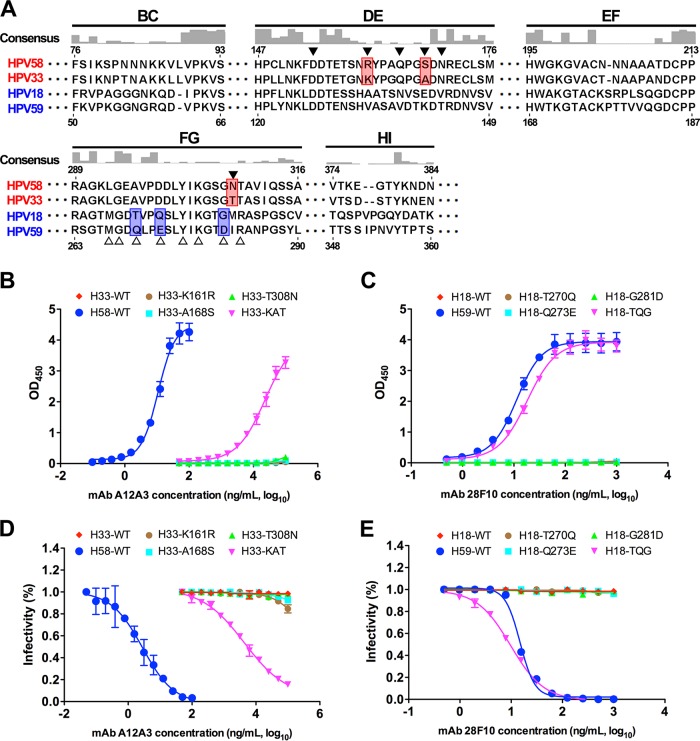
Binding and virus neutralization of type-specific neutralizing MAbs A12A3 and 28F10 can be redirected to the chimeric particles of HPV33 and HPV18, respectively. (A) Sequence alignments of L1 proteins of HPV58, -33, -18, and -59. The consensus was determined based on the alignment of L1 sequences from 36 different HPV serotypes (also see Fig. S8 in the supplemental material). The six variant regions (BC, DE, EF, FG, HI, and the C-terminal arm) are labeled. The residues involved in the antibody binding are marked by solid triangles for HPV58 and open triangles for HPV59. The different residues between HPV58 and HPV33 among the epitope residues against A12A3 are highlighted in red, and those between HPV18 and HPV59 against 28F10 are highlighted in blue. (B and C) ELISA for evaluation of binding of chimeric VLPs of HPV33 and HPV18 against MAbs A12A3 (B) and 28F10 (C). (D and E) Neutralization assay of MAbs A12A3 and 28F10 to WT and mutant PsVs of HPV33 (D) and HPV18 (E).

L1 surface loops of different HPVs exhibit a high degree of variation, which is also required for the type-specific neutralization by MAbs. This variability is the main reason for the limited cross-protection conferred by the current approved VLP-based vaccines (14, 16, 18, 19, 41–44). Notably, we found that the two type-specific MAbs A12A3 and 28F10 bind to the most variable regions of HPV58 and HPV59, respectively, based on sequence alignment of 36 HPV types (Fig. 3A and E and Fig. 7A; see Fig. S8A in the supplemental material). In the phylogenetic tree, HPV33 and HPV18 L1 sequences are closest to those of HPV58 and HPV59, respectively (Fig. S8B). To investigate whether the neutralization epitopes are involved in the determinants for type specificity, we generated a series of mutated VLPs and pseudoviruses (PsVs) of HPV33 and HPV18 by swapping one or more resides with analogous residues from HPV58 and HPV59 (Fig. 7A; Fig. S8). Swapping single residues in HPV33 (K161R, A168S, or T308N) and HPV18 (T270Q, Q273E, or G281D) did not show any binding ability to A12A3 and 28F10, the same as that seen for WT HPV33 and HPV18, respectively (Fig. 7B and C). However, both triple mutants, HPV33 KAT and HPV18 TQG, showed increased binding reactivity to A12A3 (EC_50_, 23,000 ng/ml) and 28F10 (EC_50_, 18.1 ng/ml), respectively. Similar effects on neutralization were also observed for MAbs A12A3 and 28F10 using HPV33 and HPV18 PsV mutants: the 50% inhibitory concentration (IC_50_) for A12A3 against the HPV33 KAT PsV mutant was 4,300 ng/ml, whereas no neutralizing titer was observed for A12A3 against HPV33 WT PsV (Fig. 7D). Surprisingly, levels of 28F10 neutralization of HPV18 TQG PsVs (IC_50_, 9.7 ng/ml) and HPV59 WT PsV (IC_50_, 14.9 ng/ml) were comparable (Fig. 7E). Collectively, these findings suggest that the neutralization sites of HPV58 (R161, S168, and N308) and HPV59 (Q270, E273, and D281)—pinpointed by type-specific MAbs A12A3 and 28F10—determine, at least in part, the type specificity of HPV58 and HPV59, respectively.

10.1128/mBio.00787-17.8 FIG S8  Amino acid sequence alignments of different HPV L1 proteins and the phylogeny of the L1 protein. (A) Sequence alignments of the L1 proteins from 36 different HPV serotypes. The variant regions DE and FG are labeled. The footprint residues of A12A3 (solid triangles) and 8F10 (empty triangles) are marked. (B) Phylogenetic tree of HPV L1 protein based on the amino acid sequences in panel A. The size bar presented at the bottom provides a scale for evolutionary lineages changing over time. Download FIG S8, TIF file, 2.3 MB. Copyright © 2017 Li et al. 2017 Li et al. This content is distributed under the terms of the Creative Commons Attribution 4.0 International license.

## DISCUSSION

Most of the known antigenic epitopes of HPV are situated on the five surface loops (BC, DE, EF, FG, and HI) and the C-terminal arm of HPV pentamers. This knowledge arose from the use of panels of MAbs (10, 15, 16, 18, 19, 41, 51) and from low- to medium-resolution cryo-EM structures (21, 22, 52) of HPV pseudoviruses (PsVs) in complex with several Fab fragments. Except for the U4 antibody, which binds to the invading arms between adjacent pentamers, each of the other epitope-identified antibodies adopts a top-fringe binding pattern, like MAb 28F10 in our study, with each pentamer binding five Fab fragments on the top and outside rim, regardless of any steric hindrance from neighboring pentamers. These top-fringe-binding antibodies neutralize HPV infection by preventing the virus from attaching to cell surface receptors or from undergoing further conformational changes that initiate virus endocytosis (28, 53, 54). Indeed, the 28F10 epitope overlaps with the binding sites of heparin oligosaccharides on HPV pentamers (33) (Fig. S7A and B). Thus, it is reasonable to speculate that the 28F10-bound virus would be unable to attach to this receptor and therefore fail to infect the host cell.

Very few studies, however, have identified or characterized a top-center-binding antibody that neutralizes HPV infection with one Fab fragment binding to one pentamer, as described here for A12A3, and to our knowledge, any epitope information for such an antibody is also limited in the available literature. From the crystal structure of HPV58p:A12A3, we postulate that, because the recognition sites dominantly cluster on the DE loop of the pentamer, the antibody should be able to bind to the top-center portion of the pentamer and neutralize HPV at a molar ratio of 72:1. The DE loops of an L1 pentamer only occupy a small portion of the exposed outer surface of a pentamer (13), suggesting that an antibody like A12A3 probably represents a minority of the human antibody repertoire. However, although the epitope is not immunodominant and may therefore be unable to stimulate a strong antibody response, it is plausible that an antibody like A12A3 could neutralize HPV more efficiently than a 28F10-like antibody, since a top-center mode of binding requires fewer antibody molecules to saturate the virion. The A12A3-like antibodies might block infectivity of HPV by impeding the conformational changes on the L1 capsid that are necessary for HPV infection. In addition, previous studies have shown that the minor protein L2 is located within the axial lumen of the L1 capsomers and mediates HPV infection via the exposure of its N terminus, which results in cleavage by the proconvertase enzyme furin (32, 55–57). Therefore, top-center-binding antibodies may also neutralize the virus by preventing the amino terminus of L2 from extruding to the capsid surface and subsequently blocking access to the furin recognition site. Our observations over the HPV58p:A12A3 structure indicate that the side chains of D154 and N170 (chains C, D, and E in the HPV58 pentamer) are located on the inner side of the central lumen of the pentamer and might be accessible for L2 binding (Fig. S7C and D), suggesting that these two residues may be involved in L2 interaction so that the mutation on either residue would affect virus infectivity.

Neutralization sites defined by functional antibodies, being associated with receptor binding or correlating to virus infection events, are important for understanding the early stages of virus infection (57, 58). Earlier studies using site-directed mutagenesis or loop-swapping approaches showed that the conformational and type-specific epitopes recognized by HPV neutralizing antibodies were partially mapped to the variable loops of the HPV capsid (15, 16, 18, 19, 41, 51, 53). Recent cryo-EM structural analyses of several HPV16 capsid-Fab complexes at medium resolution have shown that each of these neutralizing antibodies has a large footprint that spans three or more surface loops originating from adjacent monomers (21, 22, 52). Here, with the help of high-resolution structures, we have better defined the type-specific epitopes of two different HPV-neutralizing antibodies and shown that these antibodies recognize only 6 or 7 discontinuous amino acid residues in the virus primary sequence (Fig. 3 and 7; Fig. S8). Through structure-guided mutagenesis on HPV pseudoviruses, we further clarified exactly which epitope residues of HPV58 (D154, S168, and N170) and HPV59 (M267, Q270, E273, Y276, K278, and R283) are important for virus infection. However, the exact role of each of these residues during virus infection was not addressed in the present study. Previous studies have demonstrated that HPV entry into host cells occurs via multiple receptor engagements and conformational shifts in capsid proteins (34–40); therefore, further detailed cell-based assays recruiting proposed receptors need to be performed to decipher the biological roles of these surface residues and their interactions with cellular receptors during HPV infection.

In verifying type specificity, we used the most closely related HPV types of HPV58 and HPV59—HPV33 and HPV18, respectively—and replaced one or more residues in the VLPs and in the PsVs with the homologous residues of A12A3 and 28F10, respectively. Our results demonstrate that three residues of HPV58 (R161, S168, and N308) and HPV59 (Q270, E273, and D281) are required for type-specific antibody recognition by MAbs A12A3 and 28F10, respectively (Fig. 7). Overall, our findings point to the importance of gaining structural information not only for accurate epitope information, but also for probing the key sites for virus infection and type specificity of HPV.

## MATERIALS AND METHODS

### Ethics statement.

Experimental animals were purchased from Shanghai Institutes for Biological Sciences (Shanghai, China) and housed in the animal facility of Xiamen University Laboratory Animal Center (XMULAC). Animals were fed appropriate food and water *ad libitum*. All experimental protocols were reviewed and approved by the Animal Care and Use Committee of Xiamen University. Animal manipulation and vaccination procedures strictly adhered to the guidelines of XMULAC and were compliant with all regulations provided by XMULAC. All efforts were made to minimize suffering during vaccination, blood collection, and surgery. The Animal Ethics Committee approval number for this study was XMULAC20150200.

### Protein cloning, expression, and purification.

The coding sequences for aa 34 to 524 of HPV58 L1 protein (GenBank accession no. ADK78584.1) and aa 10 to 507 of HPV59 L1 protein (GenBank accession no. CAA54856) were cloned into the pTO-T7 vector. All of the mutated constructs were generated with site-directed PCRs. The methods for L1 protein expression and purification, the assembly of HPV L1 VLPs, and the preparations of HPV L1 pentamers were followed as described previously (10). MAbs A12A3 and 28F10 were raised from HPV58 VLP and HPV59 VLP, respectively, using a standard murine MAb preparation protocol (10, 58). Briefly, the hybridomas producing MAbs A12A3 and 28F10 were purified from mouse ascites by protein A affinity chromatography. The Fabs of A12A3 and 28F10 were obtained by papain digestion and purified with DEAE-5PW (TOSOH) exchange.

### Preparation of murine monoclonal antibodies.

BALB/c mice were purchased from Beijing Vital River Laboratory Animal Technology Co., Ltd. These animals were immunized subcutaneously three times at an interval of 2 weeks with HPV58 and HPV59 VLPs (100 µg/animal) absorbed with aluminum adjuvant. MAbs A12A3 and 28F10 were then raised using the standard hybridoma technology and screened using a pseudovirus-based neutralization assay. MAbs were produced in mouse ascites and purified by protein A affinity chromatography. The purified MAbs were subsequently diluted to 1.0 mg/ml in phosphate-buffered saline (PBS) and stored at −20°C.

### SDS-PAGE and Western blotting.

SDS-PAGE was performed using the Laemmli method with minor modifications (59). Briefly, samples were diluted with Laemmli sample buffer (0.0625 M Tris-HCl [pH 6.8], 2% [wt/vol] SDS, 10% [wt/vol] glycerol, 100 mM dithiothreitol, and 0.001% [wt/vol] bromophenol blue) to a final protein concentration of 1 or 0.2 mg/ml. Samples were heated to 80°C for 10 min, loaded into the wells of the separating gel, electrophoresed, and stained with Coomassie brilliant blue.

For Western blotting, resolved proteins were electrically transferred from the SDS gels to nitrocellulose membranes. Capsid proteins L1 and L2 were detected by enhanced chemiluminescence Western blot analysis using the monoclonal antibodies 58L1-A1H6 and L2-14H6 (60) for HPV58 pseudovirus and 59L1-27D9 and L2-14H6 for HPV59 pseudovirus. The secondary antibody was an alkaline phosphatase-conjugated goat anti-rabbit IgG.

### Indirect ELISA.

Wells of 96-well microplates were coated with wild-type or mutant VLPs or pentamers. After plate blocking (300 ng/well, incubated overnight at 4°C), 2-fold serial dilutions of the antibody were added to the wells and incubated for 1 h at 37°C. Horseradish peroxidase (HRP)-conjugated goat anti-mouse IgG antibody (diluted 1:5,000 in PBS; Abcam, Inc.; Cambridge, United Kingdom) was used to detect the antibody titers, followed by 50 µl of 3,3′,5,5′-tetramethylbenzidine liquid substrate (Sigma-Aldrich, St. Louis, MO) per well for 30 min at 37°C. The absorbance at 450 nm (reference, 620 nm) was recorded using an automated ELISA reader (Tecan, Männedorf, Switzerland). Endpoint titers were defined as the highest plasma dilution that resulted in an absorbance value 2 times higher than that of nonimmune plasma with a cutoff value of 0.05. Data are presented as log_10_ values. The median effective concentration (EC_50_ [nanograms per milliliter]) is defined as the antibody concentration for achieving 50% binding with the antigen.

### Sandwich ELISA.

The presence of conformational neutralizing epitopes for L1 of different HPV58 and -59 mutant PsVs were determined using a MAb-based sandwich ELISA. The neutralizing MAbs C2D8 and 30A1 were used as capture antibodies, with C2A1-HRP and 24F4-HRP used as detection antibodies, for HPV6 and -11, respectively. These four MAbs are all conformational neutralizing antibodies raised against HPV58 VLPs (MAbs C2D8 and C2A1) and HPV59 VLPs (MAbs 30A1 and 24F4), respectively.

### AUC.

Sedimentation velocity experiments were used to assess the molecular sizes of the Fab fragments of the antibodies, HPV L1 pentamers, and pentamer-Fab complexes at 20°C on a Beckman XL-A analytical ultracentrifuge equipped with absorbance optics and an An60 Ti rotor (Beckman Coulter, Inc.; Fullerton, CA). Samples were diluted to an optical density at 280 nm (OD_280_) of 1 in a 1.2-cm path length. The rotor speed was set to 30,000 rpm for all samples. The sedimentation coefficient was obtained using the c(s) method with the Sedfit Software (61), kindly provided by P. Schuck at the National Institutes of Health (Bethesda, MD).

### Biacore biosensor analysis.

CM-5 sensor chips were amine coupled to a goat anti-mouse antibody Fc fragment (GAM-Fc) (Biacore 3000; GE). One flow cell of a chip was coated with 13,000 resonance units (RU) of the GAM-Fc, whereas the other flow cell was left uncoated and blocked as a control. The affinity measurements of MAbs A12A3 and 28F10 binding with HPV58 and HPV59 pentamers, respectively, were initiated by passing HBS (10 mM HEPES, pH 7.4 and 150 mM NaCl) over the sensor surface for 100 s at 10 μl/min, followed by injection of 10 μg/ml of MAb A12A3 or 28F10 at 30 μl/min for 3.3 min and then injections of serially diluted antigens at 30 μl/min for 3.3 min. Every measurement on the Biacore 3000 biosensor was performed three times, and the individual values were used to produce the mean affinity constant and standard deviation.

### Crystallization and structural determinations.

An excess of purified A12A3 and 28F10 Fabs were mixed with pentamers of HPV58 and HPV59, respectively, and incubated at 37°C for 2 h for complex formation. The complexes were purified over a Superdex 200 (GE Healthcare) and concentrated to ~5 mg/ml. Crystals were grown by mixing 1 μl complex with 1 μl reservoir solution (for HPV58 pentamer, 0.2 M magnesium formate and 14.5% [wt/vol] polyethylene glycol [PEG] 3350; for HPV58:A12A3, 0.2 M lithium chloride, 0.1 M Tris [pH 8.0] and 14% [wt/vol] PEG 3350; and for HPV59:28F10, 2% tacsimate, 0.1 M sodium citrate [pH 5.5], and 17% [wt/vol] PEG 3350) using the hanging-drop vapor diffusion method at 20°C. Crystals were cryo-protected in the reservoir solution supplemented with 30% glycerol and flash-cooled at 100 K.

Diffraction data from the crystals were collected at the Shanghai Synchrotron Radiation Facility (SSRF) beamline BL17U using a Quantum-315r charge-coupled-device (CCD) area detector. All data sets were processed using the HKL-2000 program package (http://www.hkl-xray.com). Initial phases were determined by molecular replacement with PHASER (62). In molecular replacement, the search model for the HPV58 pentamer was the HPV16 L1 structure (PDB no. 3OAE); HPV58p:A12A3 was searched by two components (ensemble 1, the final HPV58 pentamer model in this study; ensemble 2, the Fab model in PDB no. 1WEJ); we searched HPV59p:28F10 using two model templates: ensemble 1, the HPV59 L1 pentamer model (PDB no. 5J6R) (10); ensemble 2, the Fab model in PDB no. 2QHR. The resulting models were manually built in COOT (10, 63), refined using PHENIX (64), and analyzed with MolProbity (65). In brief, one round of rigid-body refinement was performed after molecular replacement. After manual modification of the refined model in COOT, the coordinates and individual B factors of HPV58 pentamer were refined in reciprocal space without noncrystallographic symmetry (NCS) restraints, whereas coordinates and group B factors (one B factor group per residue) of HPV58p:A12A3 and HPV59p:28F10 complexes were refined in reciprocal space with NCS restraints and secondary structure restraints to avoid overfitting. TLS refinement was performed in the later stages with autosearched TLS groups in PHENIX, which are listed in REMARK 3 sections in deposited PDB files. Statistics for the data collection and structure refinement are summarized in Table 1.

### Sequence alignment and phylogenetic tree construction.

Multiple sequence alignment of the L1 proteins from 36 different HPV serotypes were calculated using Clustal Omega (66) on the EBI server (67). The figure indicating the sequence conservation was generated using PyMOL Molecular Graphics System, version 1.7 (Schrödinger, LLC; http://pymol.sourceforge.net). The Consurf server was used to generate the conservation scores (68). All alignments were plotted using Chimera (69). The phylogenetic tree of these 36 L1 proteins was inferred using the neighbor-joining algorithm (70) on the EBI server. Trees were visualized using FigTree (http://tree.bio.ed.ac.uk/software/figtree/).

### Cryo-EM and 3D reconstruction of HPV VLP-Fab complexes.

The VLP-Fab immune complexes were prepared by mixing HPV L1 VLPs with oversaturated Fab fragments as per the corresponding crystal structure of the pentamer-Fab complex (with molar ratios of 1:1.2 for both H58VLP:A12A3 and H59VLP:28F10). The complexes were then incubated at 37°C for 2 h. An aliquot of a 2-μl sample was deposited onto a glow-discharged Quantifoil holey carbon grid (R2/1, 200 mesh; Quantifoil Micro Tools). After 5 s of blotting to remove extra sample, the grid was plunge-frozen into liquid ethane using a FEI Vitrobot. Images were recorded on an FEI Falcon I direct detector camera at a 93,000 nominal magnification in an FEI TF30 FEG microscope at 300 kV, with underfocus settings determined to be between 1.0 and 3.0 µm using CTFFIND3 (71), and with an electron dose of ~25 e/Å^2^. Particles were manually boxed and extracted with the program Robem (72). The origin and orientation parameters for each of these particle images were estimated by means of model-based procedures (72), and an initial model was generated by the random-model method (73). After several rounds of reference-free two-dimensional (2D) and, thereafter, 3D classifications using Relion (74), good particles were selected for further 3D refinement with AUTO3DEM (72). The map resolutions were determined based on “gold standard” criteria of a 0.143 Fourier shell correlation (FSC) cutoff (75). The crystal structures were fitted into the corresponding maps using the “fit in the map” tool in UCSF Chimera (69).

### Preparation of HPV PsVs.

293FT cells for pseudovirus (PsV) production and the subsequent neutralization assay were obtained from American Type Culture Collection (ATCC). Wild-type (WT) or mutant HPV PsVs were produced as described previously (20, 76, 77). The L1/L2 expression vector and pN31-EGFP used in the experiment were kindly provided by J. T. Schiller (78). Briefly, the plasmids carrying codon-optimized WT or mutant HPV L1 genes were individually cotransfected with an L2 expression plasmid and the marker plasmid into 293FT cells. The cells were harvested 72 h after transfection, lysed with cell lysis buffer containing 0.5% Brij58 (Sigma-Aldrich), 0.2% Benzonase (Merck Millipore, Darmstadt, Germany), 0.2% PlasmidSafe ATP-Dependent DNase (Epicenter Biotechnologies, Madison, WI), and Dulbecco’s phosphate-buffered saline (DPBS)-Mg solution, and incubated at 37°C for 24 h. Afterwards, 5 M NaCl solution was added to the samples to extract the cell lysates. The 50% tissue culture infective dose (TCID_50_) of the supernatant was then measured to determine the titers of the PsVs, and the TCID_50_ values were calculated according to the classical Reed-Muench method (79). Maturation and purification of these samples followed the procedures described before (9, 77, 80).

### Neutralizing efficiency assessment of HPV antibodies.

293 FT cells were incubated at 37°C in the wells of a 96-well plate at a density of 1.5 × 10^4^ cells per well for 6 h. Neutralizing antibodies at a certain concentration were subjected to a 2-fold dilution. PsVs were diluted to 2 × 10^5^ TCID_50_/µl. Sixty microliters of the PsV diluent and 60 µl of the serially diluted neutralizing antibodies were mixed and incubated at 4°C for 1 h. The negative control was prepared by mixing 60 µl of the PsV diluent with 60 µl of the culture medium. Then, 100 µl of the mixtures described above was added to designated wells and incubated at 37°C for 72 h. Cells were then treated with trypsin and analyzed by flow cytometry. The median inhibitory concentration (IC_50_ [nanograms per milliliter]) is defined as the antibody concentration for achieving 50% inhibition of PsV.

### Accession number(s).

Atom coordinates and structure factors for the HPV58 L1 pentamer, HPV58p:A12A3 Fab, and HPV59p:28F10 Fab have been deposited in the PDB (PDB no. 5Y9E, 5Y9C, and 5Y9F, respectively). The electron microscopy (EM) density maps for HPV58v:A12A3 Fab and HPV59v:28F10 Fab have been deposited in the Electron Microscopy DataBank (EMDB) under EMD-6809 and EMD-6814, respectively.
