# Supplementary figures and images for: Crystal Structures of Two Immune Complexes Identify Determinants for Viral Infectivity and Type-Specific Neutralization of Human Papillomavirus
Source: mBio. 2017 Sep 26;8(5):e00787-17. doi: 10.1128/mBio.00787-17 (PMC5615192; doi:10.1128/mBio.00787-17)

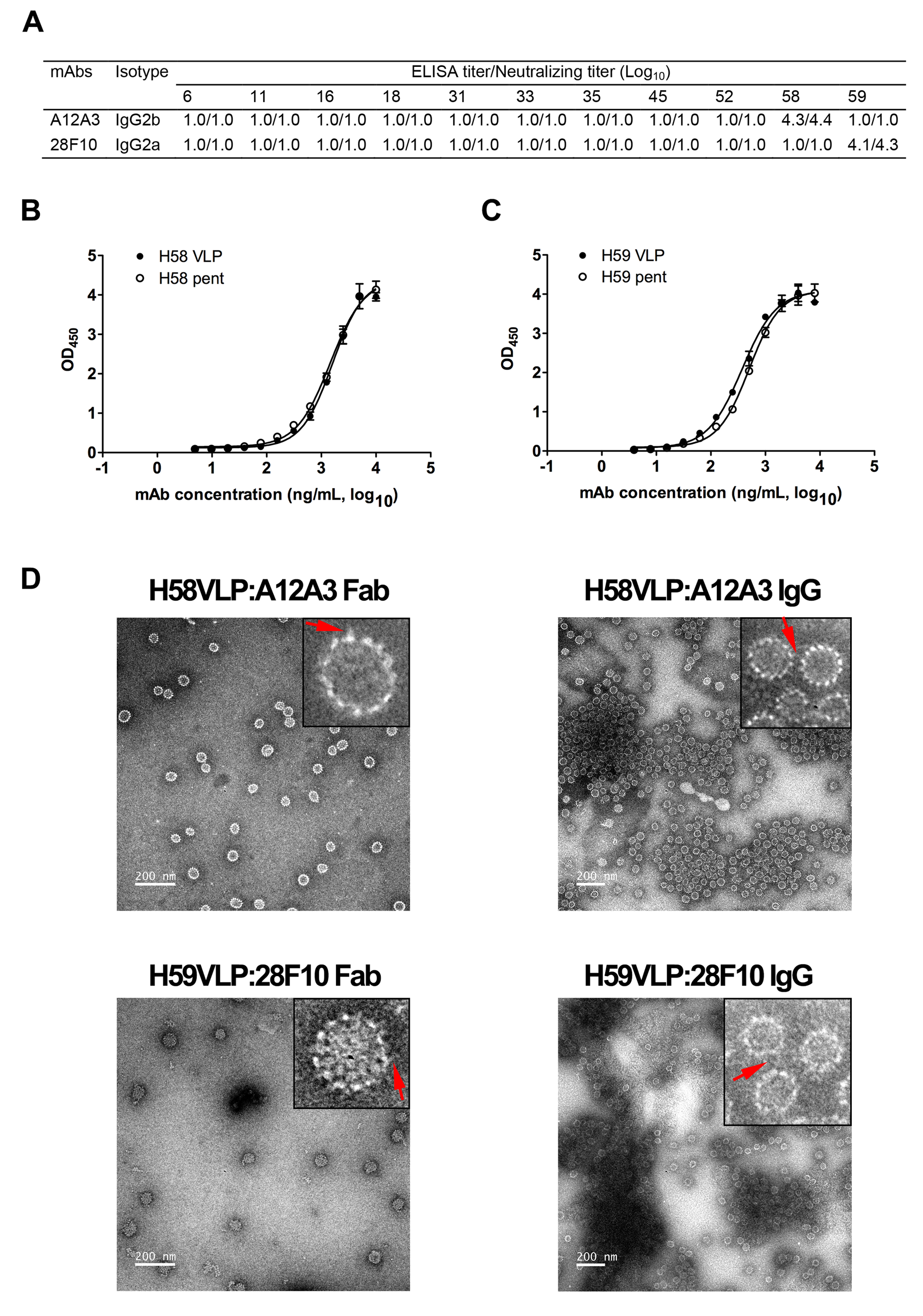

Supplement: FIG S1 [file mbo004173479sf1.tif]

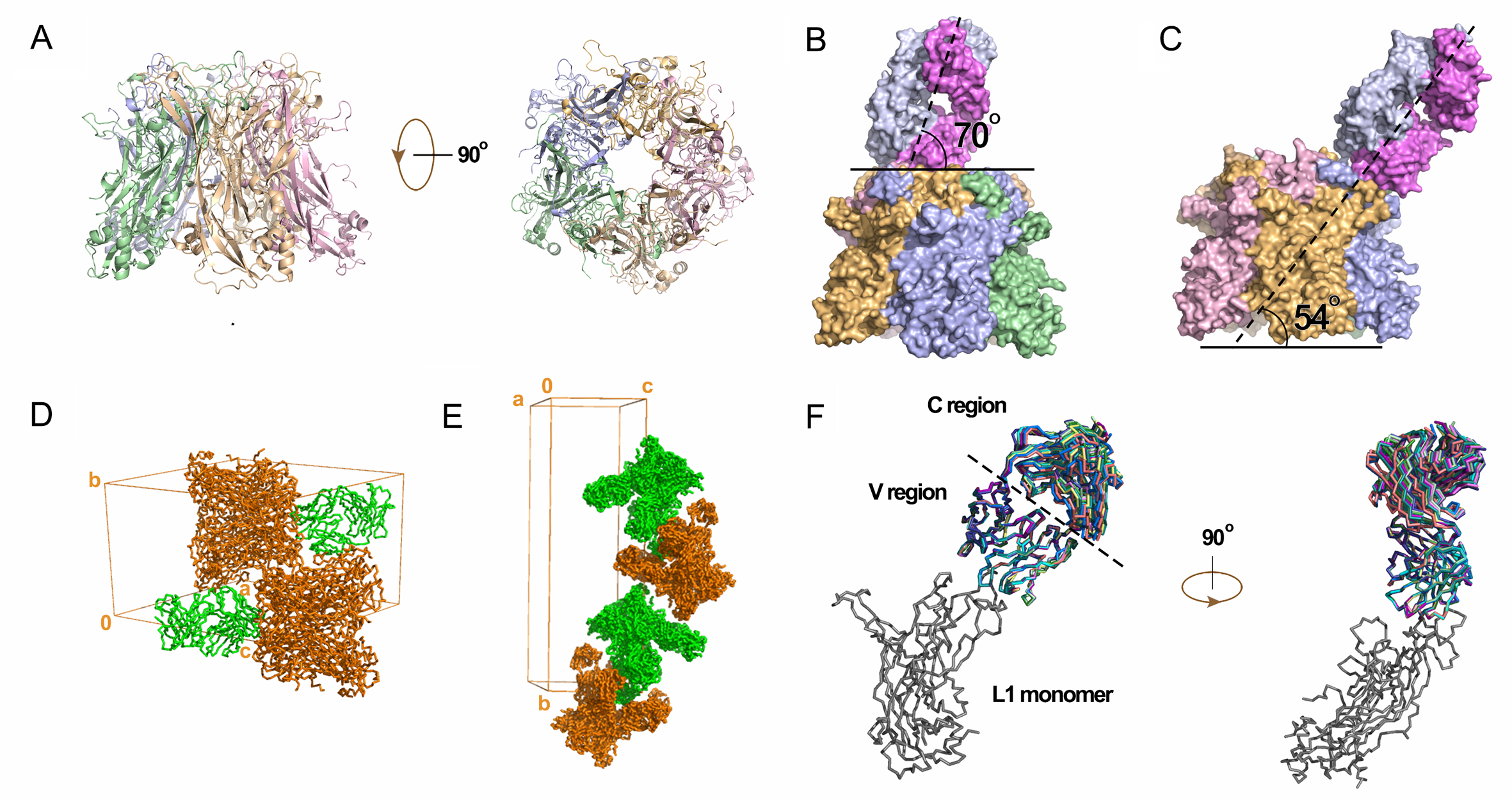

Supplement: FIG S2 [file mbo004173479sf2.tif]

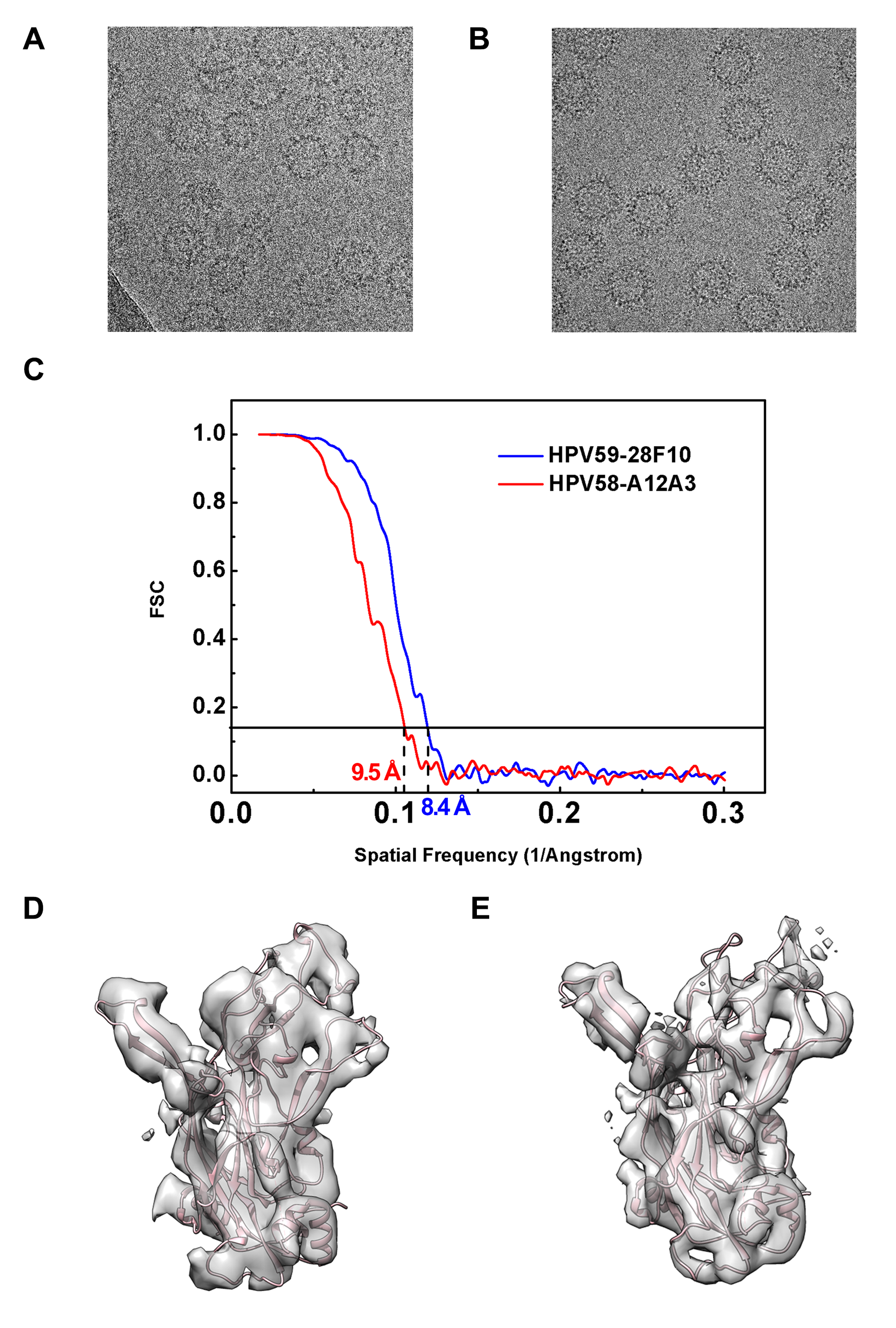

Supplement: FIG S3 [file mbo004173479sf3.tif]

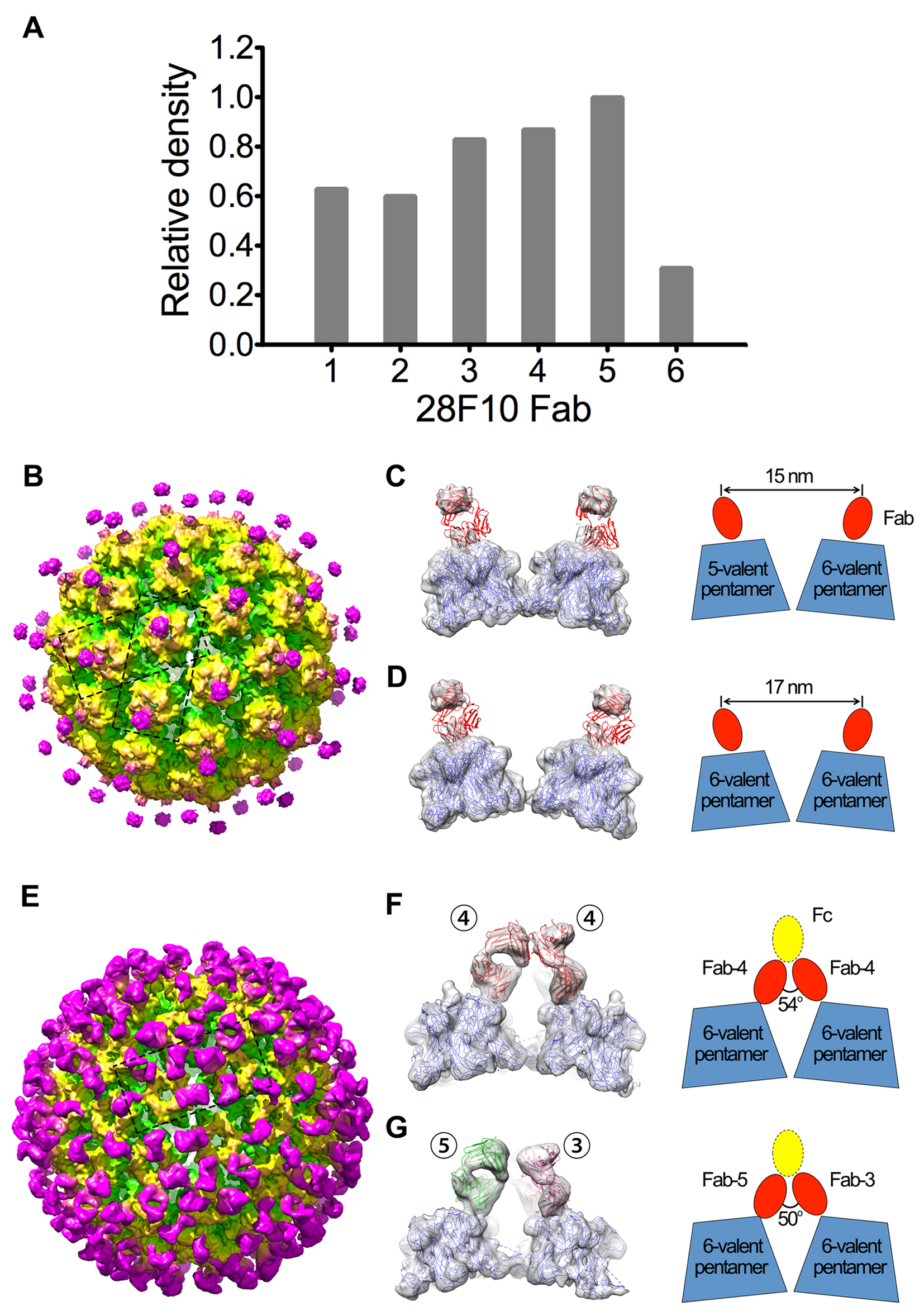

Supplement: FIG S4 [file mbo004173479sf4.tif]

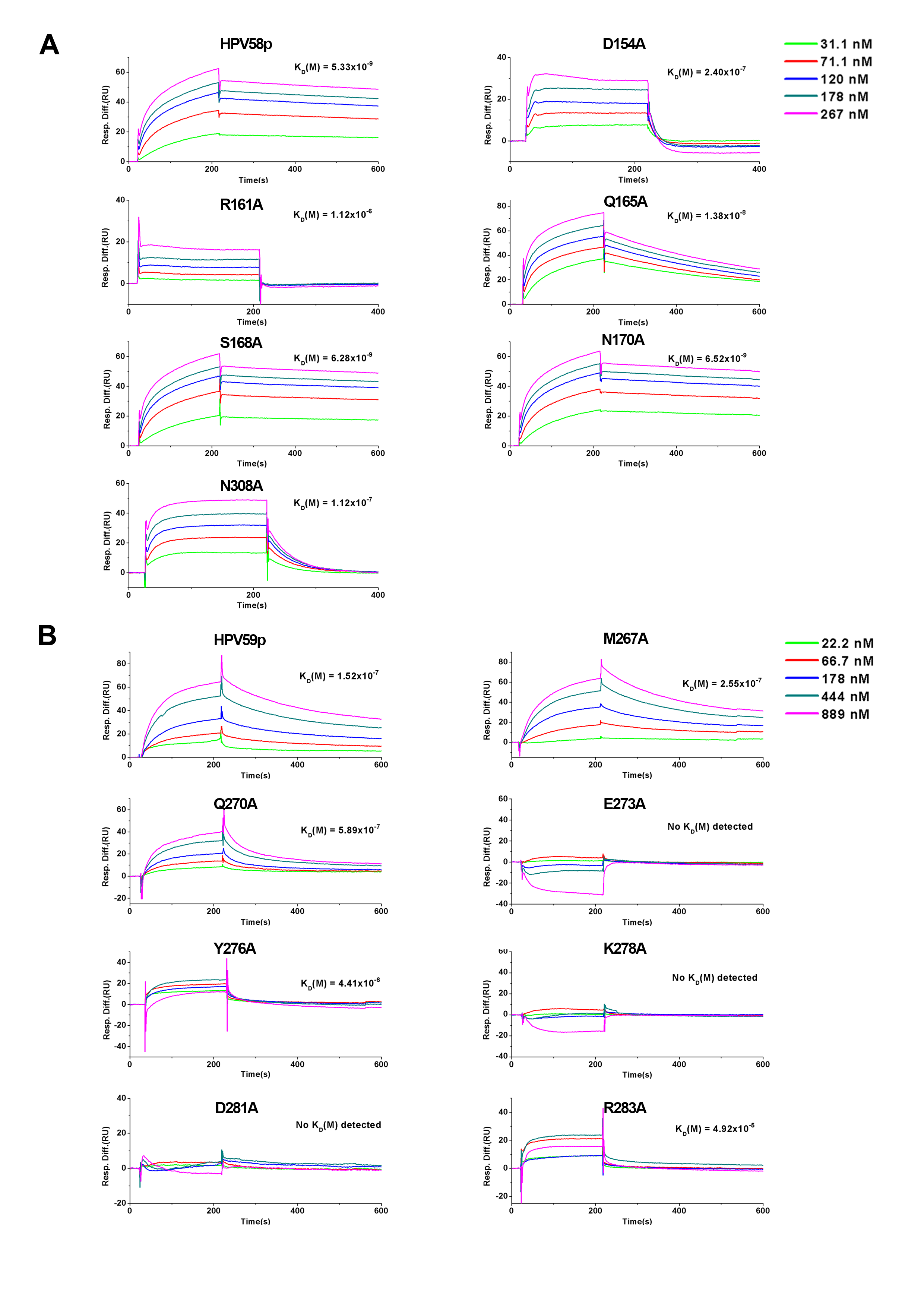

Supplement: FIG S5 [file mbo004173479sf5.tif]

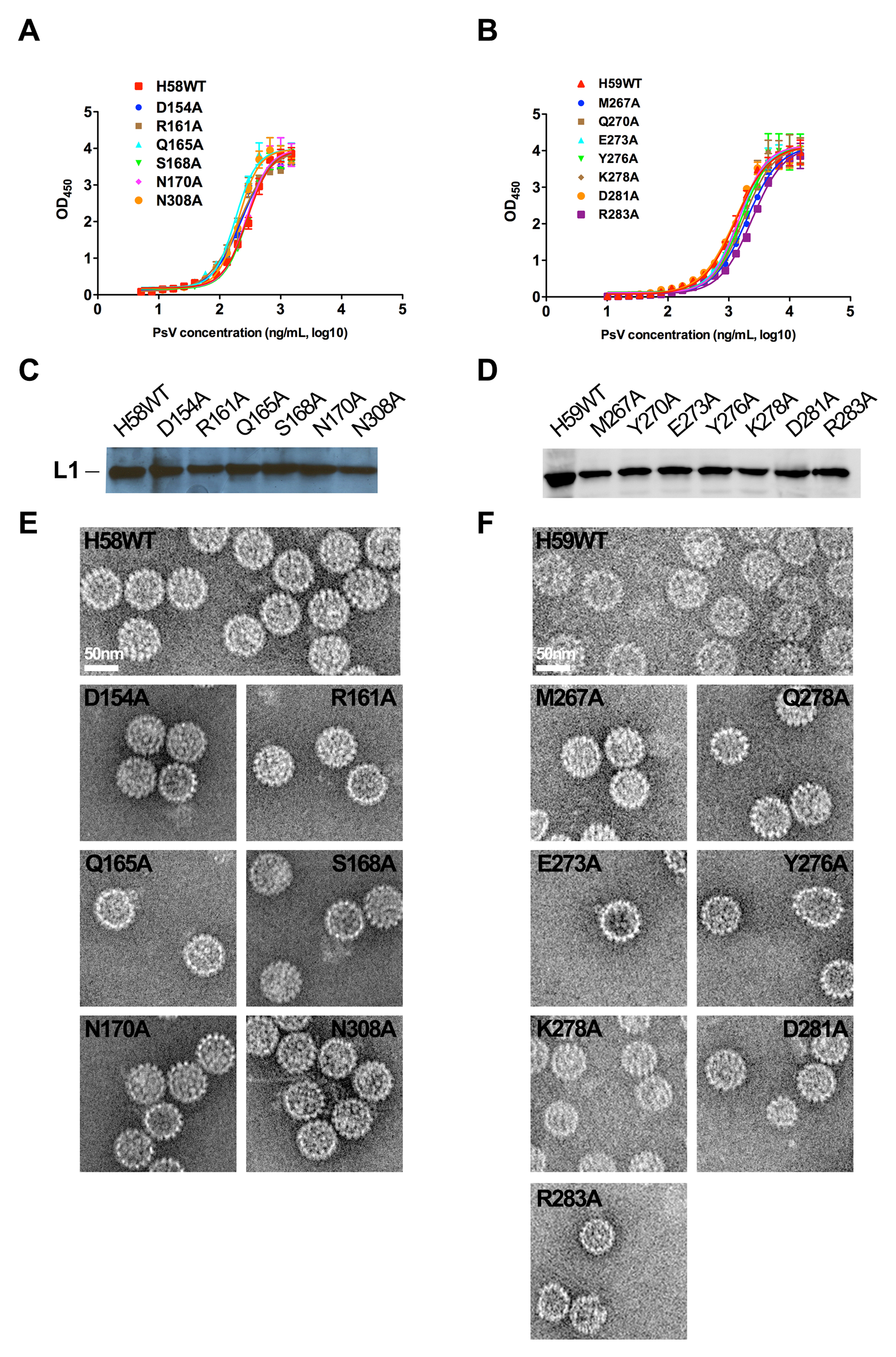

Supplement: FIG S6 [file mbo004173479sf6.tif]

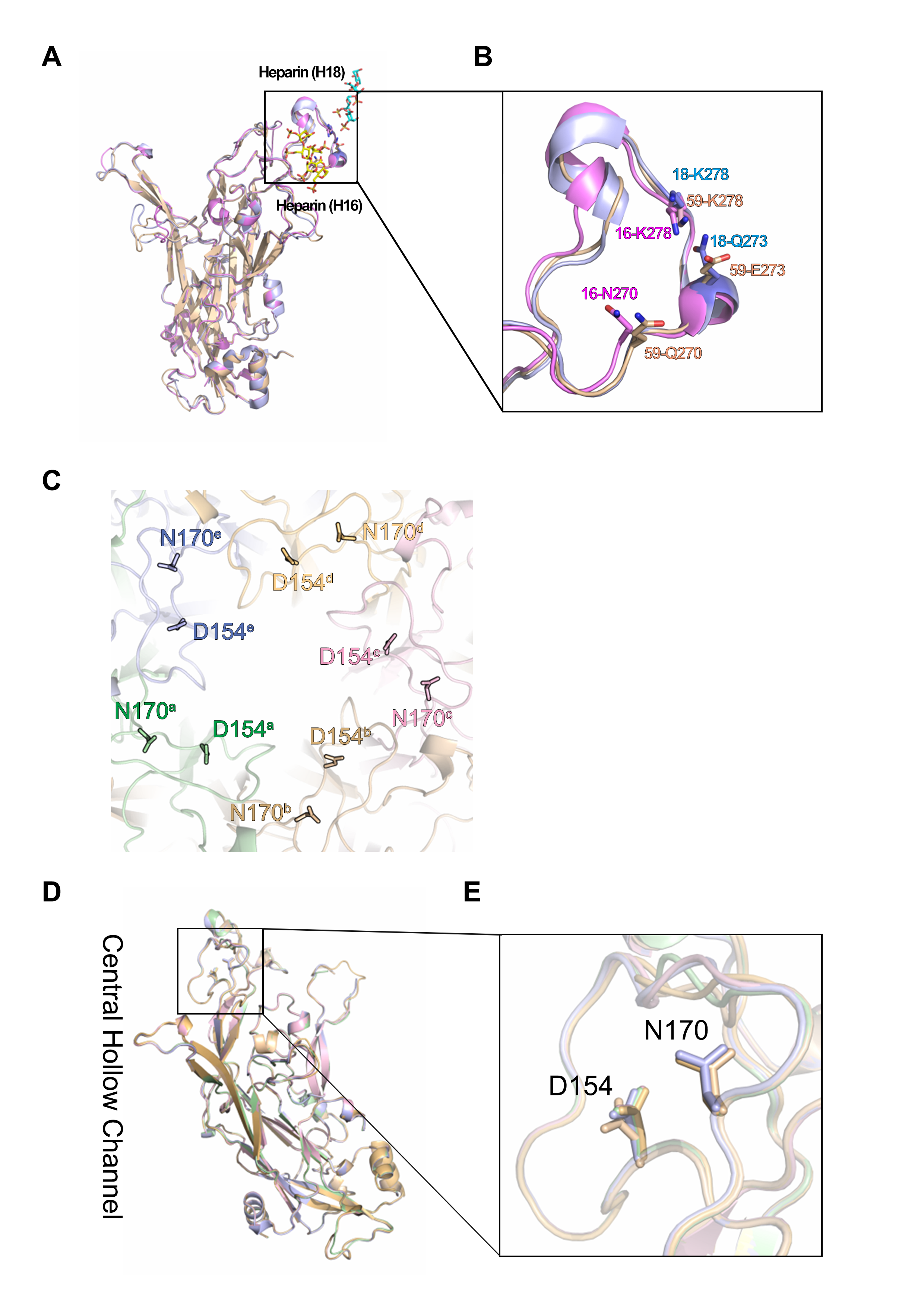

Supplement: FIG S7 [file mbo004173479sf7.tif]

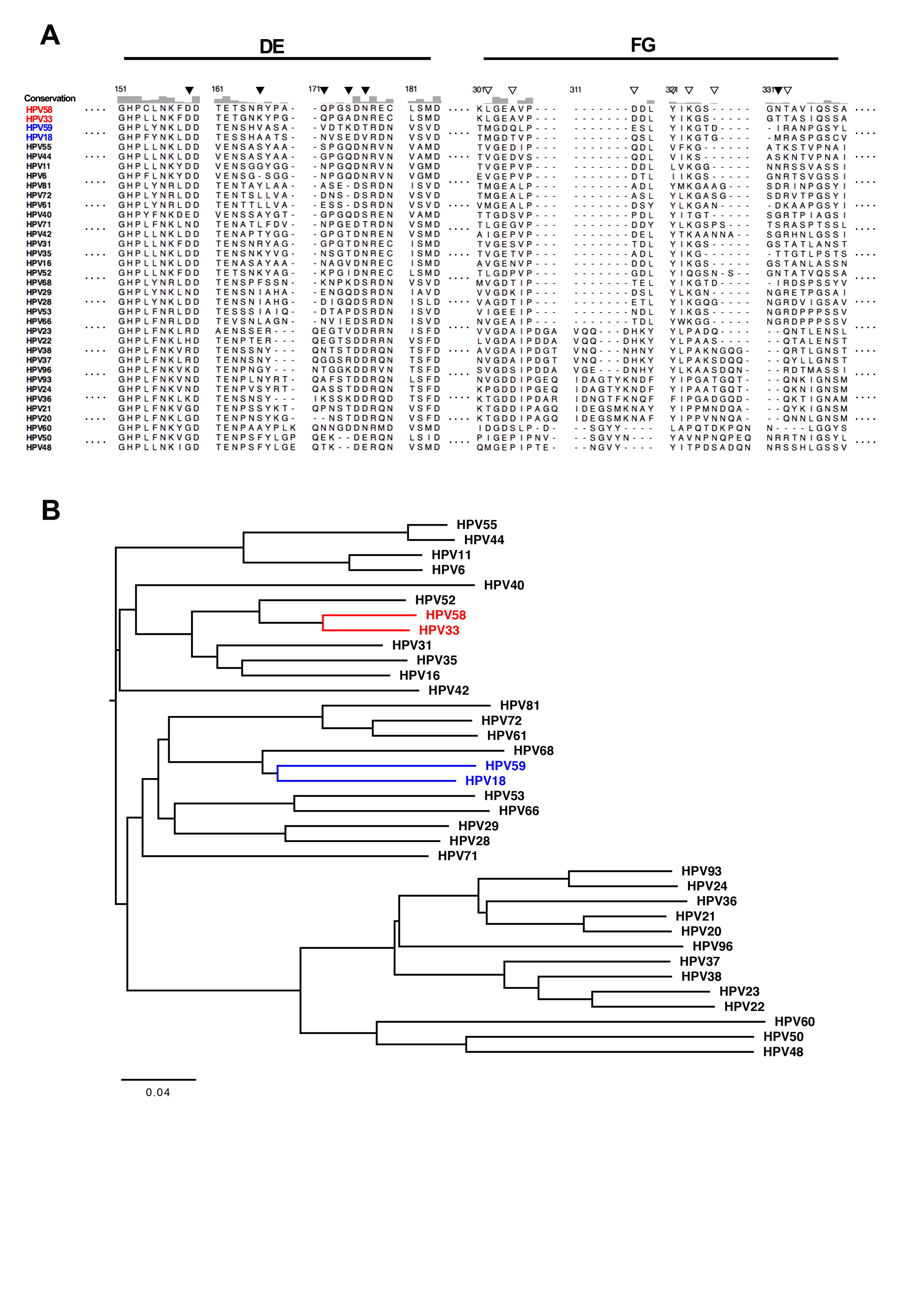

Supplement: FIG S8 [file mbo004173479sf8.tif]
