# Supplementary material for: Crystal Structures of Two Immune Complexes Identify Determinants for Viral Infectivity and Type-Specific Neutralization of Human Papillomavirus
Source: mBio. 2017 Sep 26;8(5):e00787-17. doi: 10.1128/mBio.00787-17 (PMC5615192; doi:10.1128/mBio.00787-17)
Supplement: TABLE S1 [file mbo004173479st1.docx]

**Table S1A.** Hydrogen bonding contacts between the HPV58 epitope and the A12A3 Fab.

| A12A3’s epitope on HPV58 | | A12A3 Fab residues | | Distance (Å) |
| --- | --- | --- | --- | --- |
| GLN165^a^ | (OE1) | TYR60^H^ | (N) | 3.0 |
|  | (OE1) | ARG65^H^ | (NH1) | 3.0 |
|  | (NE2) | TYR60^H^ | (O) | 3.3 |
| ASN308^a^ | (OD1) | LEU94^L^ | (N) | 3.3 |
| ASP154^b^ | (OD2) | ARG102^H^ | (NH1) | 2.5* |
| ARG161^b^ | (NH1) | ASP31^H^ | (OD1) | 2.7* |
|  | (O) | ARG102^H^ | (NH2) | 2.9 |
| GLN165^b^ | (N) | ARG102^H^ | (O) | 2.7 |
| SER168^b^ | (N) | TYR32^L^ | (OH) | 3.0 |
|  | (OG) |  | (OH) | 3.4 |
| ASN170^b^ | (O) | TYR101^H^ | (OH) | 3.3 |
|  | (ND2) |  | (OH) | 3.2 |

* This contacting specifically refers to salt bridge.

**Table S1B.** Hydrogen bonding contacts between the HPV59 epitope and the 28F10 Fab.

| 28F10’s epitope on HPV59 | | 28F10 Fab residues | | Distance (Å) |
| --- | --- | --- | --- | --- |
| MET267 | (O) | TYR102^H^ | (OH) | 2.9 |
| GLY268 | (O) |  | (OH) | 2.9 |
| GLN270 | (OE) | SER53^H^ | (OG) | 3.3 |
| GLU273 | (OE1) | THR56^H^ | (OG1) | 2.3 |
|  | (OE2) | GLY54^H^ | (N) | 3.4 |
|  | (OE2) | SER52^H^ | (OG) | 2.5 |
|  | (OE2) | SER53^H^ | (OG) | 3.1 |
| TYR276 | (O) | TYR57^H^ | (OH) | 3.4 |
| LYS278 | (N) | TYR57^H^ | (OH) | 2.9 |
| ASP281 | (OD1) | TYR105^H^ | (OH) | 3.1 |
|  | (OD2) |  | (OH) | 3.5 |
| ARG283 | (O) | ASP106H | (N) | 3.4 |
|  | (NE) |  | (O) | 3.3 |
|  | (NH2) |  | (O) | 2.8 |
|  | (NH2) | SER96^L^ | (O) | 3.1 |
