# Supplementary material for: Crystal Structures of Two Immune Complexes Identify Determinants for Viral Infectivity and Type-Specific Neutralization of Human Papillomavirus
Source: mBio. 2017 Sep 26;8(5):e00787-17. doi: 10.1128/mBio.00787-17 (PMC5615192; doi:10.1128/mBio.00787-17)
Supplement: TABLE S2 [file mbo004173479st2.docx]

**Table S2.** Binding kinetics of mAb A12A3/28F10 against HPV58p/HPV59p and mutants

| Construct | K_a_(M^-1^·s^−1^) | K_d_(s^-1^) | K_A_(M^-1^) | K_D_(nM) |
| --- | --- | --- | --- | --- |
| HPV58p | (7.84±0.28) ×10^4^ | (4.00±0.79) ×10^-4^ | (1.91±0.29) ×10^8^ | 5.33±0.85 |
| -D154A | (4.26±0.33) ×10^5^ | (1.02±0.02) ×10^-1^ | (4.18±0.25) ×10^6^ | (2.40±0.14) ×10^2^ |
| -R161A | (1.01±0.41) ×10^1^ | (9.98±0.00) ×10^-6^ | (9.94±4.05) ×10^5^ | (1.12±0.53) ×10^3^ |
| -Q165A | (1.67±0.26) ×10^5^ | (2.28±0.18) ×10^-3^ | (7.28±0.63) ×10^7^ | (1.38±0.13) ×10 |
| -S168A | (5.43±4.73) ×10^4^ | (5.22±0.62) ×10^-4^ | (1.62±0.25) ×10^8^ | 6.28±0.94 |
| -N170A | (9.84±1.00) ×10^4^ | (6.44±1.21) ×10^-4^ | (1.53±0.21) ×10^8^ | 6.52±0.78 |
| -N308A | (2.43±0.18) ×10^5^ | (2.72±0.36) ×10^-2^ | (8.99±0.57) ×10^6^ | (1.12±0.07) ×10^2^ |
| HPV59p | (2.31±0.17) ×10^4^ | (3.51±0.40) ×10^-3^ | (6.61±0.28) ×10^6^ | (1.52±0.06) ×10^2^ |
| -M267A | (1.82±0.03) ×10^4^ | (4.64±0.40) ×10^-3^ | (3.94±0.39) ×10^6^ | (2.55±0.25) ×10^2^ |
| -Q270A | (1.05±0.07) ×10^4^ | (6.16±0.55) ×10^-3^ | (1.70±0.10) ×10^6^ | (5.89±0.33) ×10^2^ |
| -E273A | N/A | N/A | N/A | N/A |
| -Y276A | (5.36±3.72) ×10^3^ | (1.75±0.17) ×10^-2^ | (3.21±2.47) ×10^5^ | (4.41±2.60) ×10^3^ |
| -K278A | N/A | N/A | N/A | N/A |
| -D281A | N/A | N/A | N/A | N/A |
| -R283A | (4.95±6.23) ×10^3^ | (7.38±5.35) ×10^-2^ | (1.38±2.14) ×10^5^ | (4.92±4.10) ×10^4^ |
